# Bench-to-Bedside Development of IMNN-001: A Non-Viral IL-12 Gene Therapy for Advanced Ovarian Cancer

**DOI:** 10.3390/pharmaceutics18101250

**Published:** 2026-10-01

**Authors:** Khursheed Anwer, Subeena Sood, Jeff Sparks, Douglas V. Faller, Ana Limon, Stacy R. Lindborg

**Affiliations:** Imunon, Inc., Lawrenceville, NJ 08648, USA; ssood@imunon.com (S.S.); jsparks@imunon.com (J.S.); dfaller@imunon.com (D.V.F.); alimon@imunon.com (A.L.); slindborg@imunon.com (S.R.L.)

**Keywords:** plasmid DNA, non-viral gene delivery, immunotherapy, IL-12, ovarian cancer

## Abstract

Interleukin-12 (IL-12) is a potent immunostimulatory cytokine with well-established antitumor activity, but systemic recombinant IL-12 administration has been constrained by inflammatory toxicity, high circulating cytokine peaks, and insufficient tumor-local exposure. IMNN-001 is a non-viral IL-12 gene therapy candidate based on the TheraPlas^®^ platform comprising an IL-12 expression plasmid formulated with synthetic polyethylene glycol–polyethyleneimine–cholesterol (PEG-PEI-cholesterol; PPC). Administered intraperitoneally, IMNN-001 is designed to enable regional transfection, localized IL-12 production for several days after a single injection, and repeatable pharmacodynamics activity within the peritoneal disease compartment. This review summarizes the rationale, formulation attributes, gene-transfer properties, pharmacodynamic durability, mechanism of action, biodistribution, toxicology, manufacturing controls, and clinical development of IMNN-001. Across preclinical and clinical studies, intraperitoneal delivery of IMNN-001 has produced preferential cytokine induction in ascites, with substantially higher IL-12 and interferon gamma (IFN-γ) levels in ascites than in blood. These effects were accompanied by immune remodeling including a favorable shift in the balance between immunostimulatory and immunosuppressive cells. In recurrent disease, activity varied by regimen and disease setting, whereas a randomized phase I/II study in newly diagnosed advanced ovarian cancer showed numerical differences in progression-free and overall survival that require confirmation in an adequately powered trial, currently in progress. Reported toxicities were predominantly local or attributable to combination therapy, although abdominal pain and catheter-related complications have been observed. An ongoing Phase III trial is evaluating whether adding IMNN-001 to standard chemotherapy improves clinical outcome. Objectives. The objective of this review is to place IMNN-001 within the broader challenge of developing safer and more effective IL-12–based cancer immunotherapies by examining how regional, non-viral gene delivery may overcome the limitations of systemic recombinant IL-12, including short circulating half-life, high systemic cytokine peaks, and dose-limiting inflammatory toxicity. Specifically, the review summarizes the IMNN-001 product rationale, formulation design, gene-transfer and transfection properties, pharmacodynamic durability, mechanism of action, immune remodeling, biodistribution, toxicology, manufacturing and stability attributes, and clinical development experience in ovarian cancer. By integrating preclinical, translational, CMC, and clinical findings, the article aims to define the scientific and clinical basis for IMNN-001 as a compartmentally targeted, durable, and repeatable IL-12 gene therapy strategy, while also positioning it within the broader landscape of emerging IL-12 delivery approaches and future therapeutic opportunities. This is a development-focused review of IMNN-001 technology and product.

## 1. Introduction

Cancer remains a leading global health challenge despite major advances in prevention, diagnosis, surgery, radiotherapy, systemic chemotherapy, targeted therapy, and immunotherapy. The most recent GLOBOCAN estimates reported approximately 20.6 million new cancer cases and 9.8 million cancer deaths worldwide in 2024, with the annual incidence projected to reach about 34.4 million cases by 2050 because of population growth and aging [1]. Although modern therapeutic approaches have improved outcomes for many malignancies, durable disease control remains limited by tumor heterogeneity, metastatic spread, treatment-related toxicity, acquired resistance to chemotherapy, targeted agents, and immunotherapy, and unequal access to effective cancer care [2,3]. These limitations have intensified interest in therapeutic strategies that can enhance antitumor activity while improving the spatial and temporal control of drug exposure, particularly approaches designed to concentrate immune activation within the tumor microenvironment and limit systemic toxicity. These unmet needs have created a rationale for gene therapy approaches that can localize and sustain therapeutic protein expression at sites of disease, including immune-modulating cytokines designed to reshape the tumor microenvironment.

Within this broader cancer burden, ovarian cancer remains a particularly important unmet need because it is frequently detected after dissemination within the peritoneal cavity and continues to cause disproportionate mortality. In the United States, approximately 21,010 new ovarian cancer cases and 12,450 deaths are projected in 2026 [4]. Worldwide, GLOBOCAN 2024 estimates indicate approximately 330,000 new cases and 214,000 deaths [1]. Prognosis is strongly stage dependent: although the overall 5-year relative survival rate in the United States is approximately 52%, it is only about 31.5% for distant-stage disease [5]. The high mortality and poor prognosis of advanced ovarian cancer therefore reinforce the need for treatment strategies that can act throughout the peritoneal disease compartment while limiting systemic toxicity.

Against this clinical backdrop, gene therapy offers a potential means of addressing advanced ovarian cancer by enabling localized and sustained expression of therapeutic proteins within the peritoneal disease compartment. More broadly, gene therapy has evolved from a conceptual approach for correcting inherited disorders into a clinically validated modality capable of delivering therapeutic proteins in vivo. Viral vectors, particularly adeno-associated virus and lentiviral systems, have enabled important clinical advances, but they also present limitations that are especially relevant when repeated or compartmentally controlled protein expression is desired. These limitations include vector-related immunogenicity, constraints on re-administration, potential tissue toxicity, limited cargo capacity for some platforms, manufacturing complexity, and, for integrating systems, both theoretical and observed concerns related to insertional mutagenesis [6,7,8,9,10,11,12,13]. These considerations have sustained interest in non-viral delivery systems that can provide flexible and regionally controlled gene expression that can re-establish a similar cytokine response after repeated dosing.

Non-viral DNA delivery approaches include chemical carriers such as cationic lipids, polymers, and dendrimers, as well as physical methods such as electroporation, microinjection, gene gun, sonoporation, and hydrodynamic injection [14,15,16,17,18,19,20,21]. Among these approaches, contemporary ionizable lipid nanoparticles (LNPs) are the most clinically advanced carriers for mRNA and have improved nucleic-acid protection, cellular uptake, and pH-responsive endosomal escape. Their performance, however, depends strongly on payload, composition, administration route, and target tissue; success with mRNA or systemic liver-directed delivery has not been successfully translated directly to large plasmid DNA or compartmental intraperitoneal administration. Compared with viral vectors, non-viral systems generally offer lower immunogenicity, reduced risk of insertional events, highly scalable manufacturing, capacity for larger plasmid constructs, and feasibility of repeat dosing [14,15,16,17,18,19,20]. Their principal challenges include lower in vivo transfection efficiency, limited cellular uptake, endosomal escape, non-permanent or transient expression reflecting non-integration, and potential carrier-related cytotoxicity [22,23,24,25]. Hence, the clinical translation of non-viral gene delivery has been uneven, reflecting persistent barriers in achieving efficient, reproducible, and therapeutically meaningful expression in vivo. Nevertheless, advances in carrier chemistry, nanoparticle design, endosomal escape, formulation stability, and biomarker-guided development continue to improve the therapeutic potential of non-viral platforms [26,27,28,29]. In oncology, these approaches may be particularly valuable when the target biology favors regional delivery, local pharmacodynamic activity, and repeated administration within an anatomically defined tumor compartment. For cytokine-based cancer immunotherapy, especially finite, multi-day or durable expression in comparison with recombinant protein after each administration may be advantageous, particularly when the therapeutic objective is to provide repeated local immune stimulation without prolonged uncontrolled systemic exposure.

This review focuses on IMNN-001, a lead clinical candidate of the TheraPlas technology platform comprising a non-viral IL-12 gene expression plasmid and a synthetic delivery system, PPC (Figure 1).

The review examines the rationale for using regional gene delivery to overcome the limitations of recombinant IL-12 protein for cancer therapy, the formulation and gene-transfer properties that support local cytokine expression, and the preclinical and clinical evidence for durable multi-day expression that can be re-established after repeated intraperitoneal administration. Biodistribution, toxicology, antitumor efficacy, clinical experience in ovarian cancer, scalable manufacturing, shelf-life, and the broader landscape of IL-12 delivery strategies designed to improve therapeutic index through spatial and temporal control of cytokine exposure are also summarized.

The distinctive contribution of this review is its integrated bench-to-bedside and product-development perspective. Two previously published reviews, including the 2019 review of IMNN-001 (formerly GEN-1/EGEN-001) that primarily addressed the rationale for IL-12 immunotherapy in ovarian cancer and the preclinical and early clinical findings then available [30], and the 2026 review focused principally on the clinical development of IMNN-001, including findings from six completed ovarian cancer trials, safety, tumor-microenvironment effects, and potential clinical positioning [31]. In contrast, the present article systematically connects the technology concept, gene carrier design, plasmid composition, nanoparticle formation and physicochemical properties, DNA protection and gene transfer, pharmacodynamic localization and duration, mechanism of action, biodistribution, toxicology, antitumor efficacy, scalable manufacturing, stability, catheter compatibility, and clinical administration with the complete development trajectory from recurrent disease to ongoing confirmatory studies. It also compares IMNN-001 with recombinant IL-12 protein, mRNA, viral, and oncolytic delivery strategies and considers unresolved developmental challenges and possible applications in other anatomically accessible tumor compartments. Thus, although some clinical evidence necessarily overlaps with the earlier reviews, the current review provides a broader development-focused framework that integrates formulation science, nonclinical pharmacology, chemistry, manufacturing, and controls, administration, and clinical evidence.

## 2. Product Rationale

The rationale for IMNN-001 is anchored in a central pharmacologic objective to achieve localized IL-12 delivery, sustain biologically active expression over a clinically relevant interval, and permit repeat administration to maintain intraperitoneal immune stimulation. Recombinant IL-12 protein therapy has historically been limited by rapid systemic exposure, high peak cytokine levels, and inflammatory toxicity, while often failing to maintain adequate cytokine exposure within the tumor microenvironment [32,33,34,35,36]. In contrast, regional plasmid-based delivery can support in situ IL-12 production by cells within or near the tumor compartment, thereby aligning cytokine exposure with disease anatomy. IMNN-001 uses a non-viral gene therapy formulation to generate transient multi-day IL-12 expression after each administration, with the potential for repeated dosing without the anti-vector immunity and re-dosing constraints associated with many viral platforms [6,7,8]. This design is particularly suited to advanced ovarian cancer, where tumor deposits, ascites-associated cells, and immune effector cells are distributed throughout the peritoneal cavity and can be reached by intraperitoneal administration.

## 3. Composition and Formulation Attributes

IMNN-001 consists of the IL-12 expression plasmid phIL-12-005 formulated with the PPC delivery system in isotonic lactose [37]. Subsequent lyophilization results in a dry powder as the final drug product form that is later hydrated at the time of administration [38]. The plasmid expression cassette contains the elements required for functional human IL-12 expression, including separate *p35* and *p40* IL-12 genes under control of cytomegalovirus immediate-early enhancer/promoter sequences and a human growth hormone (*hGH*) 3′ untranslated region (3′UTR) containing the polyadenylation signal, within a kanamycin-resistance plasmid backbone (Figure 2) [30]. The kanamycin-resistance gene is used solely for bacterial selection during phIL-12-005 manufacture and might potentially pose a theoretical horizontal-transfer risk. This risk is mitigated through extensive plasmid purification and release testing, although it cannot be eliminated entirely. Importantly, IMNN-001 has progressed through multiple clinical studies into an ongoing Phase 3 trial, with no evidence of horizontal transfer reported during its clinical development.

The gene delivery component of IMNN-001 is PPC, a synthetic polymer based on polyethyleneimine (PEI; 2000 Da) covalently modified with cholesterol and polyethylene glycol (PEG; 550 Da) (Figure 3) to improve delivery efficiency while limiting cytotoxicity [37,38]. The target molar grafting ratios are 1.9 mol of PEG per mol of PEI, with an acceptable range of 1.4–2.4, and 0.7 mol of cholesterol per mol of PEI, with an acceptable range of 0.5–0.9. The approximate molecular weight of PPC is 5 kDa. The PEI backbone promotes gene delivery by condensing DNA into compact nanostructures and facilitating endosomal escape [37,39,40,41,42], thereby enhancing intracellular trafficking. Because both transfection efficiency and toxicity increase with PEI molecular weight [43,44], the PPC carrier in IMNN-001 was designed using low-molecular-weight PEI (PEI_2000_).

Cholesterol conjugation imparts amphiphilic properties that enhance interaction with cellular membranes, improve DNA condensation, increase cellular uptake, and promote endosomal escape, resulting in greater transfection efficiency than unmodified PEI in vitro [45,46,47]. These effects likely reflect both hydrophobic interactions with lipid bilayers and receptor-mediated endocytosis through the LDL-receptor pathway [48]. Together, cholesterol and PEG modifications increase cytoplasmic DNA availability, improve serum stability, and enhance overall gene expression.

PEGylation of the low-molecular-weight PEI_2000_-cholesterol carrier was introduced to improve nanoparticle stability and in vivo performance. PEG can reduce nonspecific protein adsorption, aggregation, and recognition by the mononuclear phagocyte system, effects that are particularly important for cationic PEI-based complexes [49,50,51,52]. Optimization of the PEG:PEI:cholesterol ratio in preclinical models showed that incorporation of approximately two PEG molecules per PEI-cholesterol unit produced the highest luciferase expression, approximately 10-fold greater than the PEI-cholesterol polymer without PEG [37]. These findings supported the PPC design as a carrier intended to balance DNA condensation, cellular uptake, expression efficiency, tolerability, and repeatable administration.

IMNN-001 nanoparticles are produced by combining plasmid DNA and PPC at defined concentrations in isotonic lactose under controlled formulation conditions. The resulting complexes are characterized by particle size, zeta potential, DNA protection from nuclease degradation, and gene-transfer activity [37]. Fully condensed IMNN-001 nanoparticles are 140 + 30 nm and a zeta potential of about +22 mV at a nitrogen to phosphate (N:P) ratio of 11:1 at which the DNA is fully condensed.

## 4. Gene Transfer and Transfection Properties

As shown in Figure 4A, the plasmid gene transfer activity of DNA plasmid was significantly (*p* < 0.05) increased following complexation with PPC at nitrogen-to-phosphate (N:P) ratios of 5 or greater compared with unformulated plasmid DNA. Optimal gene transfer was observed at N/P ratios above 10:1, where plasmid DNA was fully condensed by the polymer, as reflected by a reduction in particle size, positive zeta potential, and absence of detectable free DNA [37]. Although expression increased modestly between ratios of 11:1 and 20:1, evidence of cytotoxicity at 20:1 supported routine use of the 11:1 ratio, which maintained gene-transfer activity without detectable cell toxicity. Plasmid was formulated at an N/P ratio of 11:1.

Protection of plasmid DNA is a critical determinant of non-viral gene transfer. Complexation of plasmid DNA with PPC protected it from DNase-mediated degradation, as demonstrated by gel electrophoresis of DNA recovered after DNAse incubation and subsequent nanoparticle decomplexation with dextran sulfate (Figure 4B, right panel), whereas unprotected plasmid DNA was degraded under the same conditions (Figure 4B, left panel). This protective effect is relevant in biological fluids and tissues, where naked plasmid DNA is exposed to extracellular nucleases and, after uptake, to endosomal and lysosomal degradation [53,54,55].

Although PPC protects plasmid DNA from extracellular and intracellular degradation during delivery, the plasmid must subsequently be released from the positively charged complex to support gene expression. This release process has not been directly characterized for IMNN-001. Literature on other PEI–DNA polyplexes suggests that release is unlikely to result from a single trigger. Competitive interactions with intracellular polyanions, including glycosaminoglycans, proteins, RNA, and phosphate-containing metabolites, may weaken polymer–DNA association. Consistent with this principle, dextran sulfate can experimentally decomplex IMNN-001 nanoparticles, and heparin has been shown to induce dissociation of PEI–DNA complexes [56]. However, the extent of polyanion-induced dissociation is formulation dependent, and heparan sulfate may adsorb to some cationic polyplexes without causing complete disassembly [57].

### Potential Mechanism of PPC-Mediated Delivery

Mechanistically, the complementary functions of the PPC components are expected to act sequentially during IL-12 plasmid delivery. PPC first condenses and protects the plasmid; the resulting cationic, cholesterol-containing nanoparticles then associate with cell membranes and enter through endocytic pathways. Endosomal acidification may increase PEI protonation and alter polymer conformation, particle organization, and membrane interactions, although these effects have not been demonstrated experimentally for IMNN-001. Together with cholesterol-mediated bilayer association, these changes may promote endosomal destabilization and cytoplasmic access, although the classical proton-sponge model remains debated and is unlikely to be the sole escape mechanism [40,41,42]. In the cytoplasm, intracellular polyanions, ionic conditions, pH-dependent conformational changes, and particle dilution may weaken PPC–DNA association and facilitate plasmid release for nuclear trafficking and IL-12 expression [56,57]. However, nuclear colocalization of nanoparticles containing the PPC carrier in the biodistribution study suggests that intact or incompletely dissociated nanoparticles may also reach the nucleus. Thus, PPC-mediated delivery likely involves multiple, nonexclusive pathways; however, uptake, endosomal escape, decomplexation, and nuclear entry have not been directly quantified for IMNN-001. Product-specific studies will be informative in characterizing DNA decomplexation under relevant pH, ionic-strength, and intracellular-polyanion conditions.

PPC was selected during the early development of IMNN-001 because its components are designed to provide these complementary functions for plasmid condensation, protection, cellular uptake, intracellular trafficking, and gene expression. Along with these mechanistic attributes, practical product-development considerations—including scalable manufacture, lyophilized-product stability, catheter compatibility, feasibility of repeated intraperitoneal administration, and accumulated clinical experience supported the continued development of PPC. These formulation, manufacturing, and administration characteristics are described in greater detail in subsequent sections.

Within this product-specific context, contemporary ionizable lipid nanoparticles provide an important comparator. These systems promote pH-responsive endosomal escape and have achieved substantial clinical validation, particularly for RNA delivery [28,29]. Plasmid DNA, however, additionally requires nuclear entry, and nanoparticle performance depends strongly on payload, formulation composition, administration route, and target tissue. Because PPC and modern lipid nanoparticles have not been compared directly using the same plasmid payload, route, dose, and disease model, their relative transfection efficiency, pharmacokinetics, toxicity, repeat-dose feasibility, and manufacturability remain unknown. PPC should therefore be viewed as a carrier supported by the accumulated IMNN-001 development experience, rather than as demonstrably superior to contemporary lipid-nanoparticle technologies.

## 5. Local, Durable, and Repeatable IL-12 Delivery

The defining therapeutic concept of IMNN-001 is local delivery of IL-12 gene-encoding plasmid and consequent local expression of IL-12 protein that lasts for several days and is reproducible after repeatable administration. Intraperitoneal administration is intended to concentrate IL-12 activity within the peritoneal tumor compartment while limiting systemic cytokine exposure. This profile addresses key limitations of recombinant IL-12 protein therapy, including short half-life, high systemic peak concentrations, exaggerated downstream cytokine release, and dose-limiting inflammatory toxicities. By enabling cytokine production within the tumor-bearing compartment, IMNN-001 is designed to provide sustained immune stimulation at the site of disease without the systemic exposure typically associated with parenteral recombinant cytokine administration.

Evidence from both preclinical and clinical studies in ovarian cancer indicates that intraperitoneal administration of IMNN-001 enables local delivery of IL-12 plasmid and protein that lasts for several days with minimal systemic exposure [30,31,58,59,60,61,62]. In a mouse model of peritoneally disseminated ovarian cancer, intraperitoneal administration of a murine IMNN-001 (mIMNN-001) formulation containing a mouse IL-12 plasmid and the PPC carrier produced a several-fold increase in IL-12 levels in the tumor ascites [58]. This increase was accompanied by a concomitant rise in IFN-γ. Levels of both cytokines were approximately 10-fold higher in ascites than in serum, indicating that cytokine induction remained largely confined to the peritoneal cavity [58]. The durability and repeatability of this response were further supported by serial dosing experiments (Figure 5). Increases in murine IL-12 and IFN-γ persisted for several days after administration and followed similar kinetics after repeated treatment, indicating that weekly IMNN-001 dosing can reinforce local cytokine production without apparent loss of pharmacodynamic responsiveness. These findings are central to the IMNN-001 design to deliver not a single cytokine pulse, but rather to create a repeatable pattern of compartmental immune activation that can be maintained over successive administrations.

Clinical studies in recurrent and newly diagnosed advanced ovarian cancer likewise showed that IMNN-001 delivers IL-12 locally for several days after intraperitoneal administration [38,60,61,62]. In the first-in-human monotherapy study, escalating intraperitoneal doses of IMNN-001 (0.6–24 mg/m^2^) increased IL-12 and IFN-γ levels in ascites but not in blood. Plasmid DNA was also concentrated in ascites, where plasmid levels were approximately 10^4^- to 10^6^-fold higher than in blood (Figure 6A) [38]. Local cytokine increases were also observed in a subsequent study of IMNN-001 plus standard of care chemotherapy in platinum-sensitive ovarian cancer patients [60]. In a subsequent study of IMNN-001 combined with pegylated liposomal doxorubicin (PLD) in recurrent ovarian cancer, ascitic IL-12 rose from undetectable levels to a peak mean of 65 ± 46.3 pg per 100 mg protein, while IFN-γ and tumor necrosis factor-α (TNF-α) increased 46-fold and 13-fold, respectively [61]. By contrast, plasma cytokine changes were minimal (<2-fold), further supporting a localized pharmacologic effect. After a single treatment, ascitic IL-12 peaked within 4 days and remained above baseline through day 7 (Figure 6B), demonstrating a persistent response compared with recombinant IL-12 therapy [61]. In patients with newly diagnosed advanced ovarian cancer, intraperitoneal IMNN-001 (36 and 79 mg/m^2^) given with standard neoadjuvant chemotherapy produced dose-dependent increases in ascitic IL-12 and IFN-γ, ranging from 3.2- to 23-fold for IL-12 and 3.1- to 67-fold for IFN-γ (Figure 6C,D) [62]. The corresponding increases in blood were much smaller, ranging from 1.2- to 3.1-fold. Together, these studies show that IMNN-001 enables a delivery profile where IL-12 is produced locally at tumor site for several days which is reproduced upon repeated administration, consistent with the product’s design rationale.

The clinical dosing schedule has been guided by the observed kinetics of cytokine induction after intraperitoneal administration. In a mouse model of peritoneally disseminated ovarian cancer, alternative dosing intervals were explored to determine whether less frequent administration could preserve antitumor activity [63]. IMNN-001 expressing murine IL-12 was administered weekly, every two weeks, or every three weeks, with translational samples collected after comparable treatment exposure and remaining animals followed for tumor burden and survival. Tumor progression and mortality were broadly comparable between the weekly and bi-weekly dosing schedules, and both regimens performed better than every three-week dosing (Figure 7). These findings suggest that durable local pharmacodynamic activity lasting for several days may allow flexibility in dosing frequency. These data reinforce the concept that repeatable IL-12 delivery by IMNN-001 can be adjusted to sustain local immune activation while potentially reducing treatment burden.

## 6. Mechanism of Action and Immune Remodeling

IL-12 is among the most potent anticancer cytokines because it connects innate and adaptive immunity and can shift an immunosuppressive tumor microenvironment toward a type 1 inflammatory state. As shown in Figure 8, IL-12 activates and expands natural killer cells and cytotoxic CD8^+^ T cells, enhances IFN-γ production, supports Th1 polarization, promotes dendritic-cell maturation and antigen presentation, and can indirectly suppress tumor angiogenesis [65,66,67,68].

For IMNN-001, the therapeutic premise is that localized intraperitoneal expression of IL-12 can reproduce these immune-stimulatory effects within the peritoneal tumor compartment while limiting systemic cytokine exposure. Across preclinical and clinical studies, IMNN-001 has been associated with immune changes consistent with localized IL-12 pharmacology [30,58,60,61,62,63,64]. In the OVATION I study, increases in ascitic IL-12 and IFN-γ after IMNN-001 plus neoadjuvant chemotherapy were accompanied by reductions in tumor-associated immune-regulatory markers, including Foxp3, IDO1, PD-1, and PD-L1, in a majority of evaluable patients, with greater reductions observed in the high-dose group than in the low-dose group (Figure 9) [62]. Ratios of CD8^+^ cells to these suppressive markers increased in most patients, with statistically significant increases reported for CD8^+^/Foxp3 and CD8^+^/PD-1. Paired tumor analyses also showed increased CD8^+^ T-cell density, reduced CD4^+^ T-cell density, and an increased CD8^+^/CD4^+^ ratio after treatment [62]. These ratios reflect the balance between CD8^+^ T-cell infiltration and immune-regulatory markers, not CD8^+^ T-cell function. In ascites, favorable increases were observed in myeloid dendritic-cell and effector-memory T-cell populations. These findings suggest that repeated local IL-12 expression can remodel both the tumor and peritoneal immune compartments toward a more cytotoxic, immune-stimulatory phenotype.

Preclinical immune-profile studies provide mechanistic support for these clinical observations. After a single intraperitoneal administration of murine IMNN-001, cytokine and gene-expression analyses of ascites and tumor nodules 1 day after treatment in tumor-bearing mice showed preferential induction of Th1-associated transcripts and downregulation of selected Th2- and Th17-associated signals [58]. IL-12 was among the most strongly upregulated transcripts, and the immunomodulatory pattern was more pronounced in ascites than in tumor nodules, consistent with broad exposure of cells within the peritoneal compartment and localized transfection of tumor-associated and host immune cells. Collectively, these data support a mechanism in which repeatable regional IL-12 expression promotes IFN-γ-driven immune activation, enhances effector-cell function, and reduces suppressive pathways that otherwise limit antitumor immunity in ovarian cancer.

## 7. Nonclinical Biodistribution

A GLP biodistribution study evaluated the tissue distribution and clearance of IMNN-001 plasmid DNA after single subcutaneous or intraperitoneal administration in male and female CD-1 mice, with sampling at 3, 30, and 88 days [59]. This study examined whether plasmid exposure remained predominantly local after regional administration and whether systemic distribution persisted over time. No test article-related effects were observed on mortality, clinical signs, body weight, or food consumption.

After subcutaneous injection, systemic exposure was low, with the greatest amount of plasmid DNA remaining at the injection site. After intraperitoneal dosing, plasmid DNA was detectable across examined tissues but was concentrated primarily locoregionally in organs and tissues including gonads, liver, mesenteric lymph nodes, and spleen. Considerably lower levels were detected in blood, heart, femur marrow, and brain. The mechanisms underlying tissue exposure may include direct peritoneal contact, regional lymphatic drainage, and systemic absorption and may differ among tissues and sexes. Clearance was substantial: approximately 95–98% of plasmid DNA was cleared within about 1 month, and more than 99% was cleared by approximately 3 months. These findings support the concept that IMNN-001 provides regional exposure with progressive clearance rather than long-term systemic persistence.

Within this overall pattern of predominantly locoregional distribution and progressive clearance, the gonadal findings warrant more detailed consideration because of their potential relevance to germline risks. Intraperitoneal dosing resulted in gonadal exposure, with Day-3 concentrations reaching 79,497–873,622 copies/µg tissue DNA in males and from not quantifiable to greater than 1,000,000 copies/µg in females. Gonadal plasmid levels declined markedly by Day 30 and were at or near assay limits by Day 88, with maximum values of 704 copies/µg in males and 460 copies/µg in females. After subcutaneous dosing, gonadal plasmid DNA was below the limit of detection on Days 3 and 30, whereas the injection site contained a high and more persistent local plasmid depot through Day 88. Whole-tissue plasmid detection demonstrates gonadal exposure but does not establish germ-cell uptake or genomic integration; therefore, rare integration and inadvertent germline transmission remain theoretical risks. Conventional plasmid DNA lacks active integration mechanisms and remains predominantly episomal, giving it a substantially lower insertional mutagenesis risks than integrating viral vectors, but lack of an active integration mechanism does not completely exclude potential risks. Tissue-specific studies distinguishing episomal from integrated DNA and targeted reproductive-tissue evaluation would further define the theoretical risks of insertional mutagenesis and germline transmission. In the current primary indication under development, advanced ovarian cancer, potential reproductive risks associated with IMNN-001 are likely to have limited clinical relevance because the treated population is generally not expected to retain reproductive potential. 

This biodistribution study quantified plasmid DNA and did not directly measure the PPC carrier; consequently, the progressive clearance described above does not represent carrier clearance. However, given the relatively low molecular weight of PPC (<10 kDa), renal elimination may contribute to its clearance; this possibility remains to be confirmed through direct carrier-specific pharmacokinetic and excretion studies.

Cellular uptake studies further support the local mechanism of action. Light microscopy of ascites-derived cells from animals administered fluorescently labeled PPC complexed with murine IL-12 plasmid showed uptake by both green fluorescent protein (GFP)-expressing ID8 tumor cells and non-tumor cells present in hemorrhagic ascites (Figure 10) [58]. GFP-positive tumor cells appear green, labeled nanoparticles appear red, and DAPI-stained nuclei appear blue. The left panel shows nanoparticle colocalization with GFP-expressing tumor cells, whereas the right panel shows nanoparticles in non-tumor cells and a GFP-expressing tumor cell without detectable nanoparticles, indicating uptake by both tumor and host cells. These findings indicate that tumor-associated and host cells within the peritoneal fluid can internalize the formulation and may contribute to local IL-12 expression after intraperitoneal dosing.

## 8. Nonclinical Toxicology

A 28-day GLP toxicology study assessed weekly subcutaneous or intraperitoneal administration of mIMNN-001 in male and female CD-1 mice, followed by a 28-day recovery period to evaluate persistence, reversibility, or delayed onset of findings [69]. Animals received control article or mIMNN-001 DNA dose at 10, 50, or 250 µg/animal once weekly on Study Days 1, 8, 15, and 22. These doses corresponded to approximately 1.2, 6.0, and 30.0 mg/m^2^ and were selected before the initiation of human clinical trials to span the anticipated clinical dose range. In-life assessments included clinical observations, body weight, and food consumption, followed by clinical pathology, gross necropsy, organ evaluation, and histopathology.

The study showed no compound-related deaths, no treatment-related clinical signs, and no adverse effects on body weight or food consumption. Gross necropsy, organ weights, hematology, coagulation, and clinical chemistry revealed no clinically meaningful systemic toxicity after either intraperitoneal or subcutaneous administration. This safety profile is consistent with the intended pharmacologic design of IMNN-001 for local immune activation after regional delivery without substantial systemic toxicity.

Histopathology identified dose-related local inflammatory changes, most notably granulomatous inflammation on pericapsular, serosal, and mesenteric surfaces of organs within or adjacent to the peritoneal cavity after intraperitoneal administration. The most prominent finding was mild inflammation on the liver serosal surface at the highest dose. After subcutaneous administration, mild granulomatous to pyogranulomatous inflammation occurred at the injection site. These findings are consistent with the expected local pharmacologic activity of IL-12 and the inflammatory properties of a regional immune-stimulatory therapy.

Importantly, inflammatory changes diminished after the recovery period, indicating reversibility. Based on these findings, the no-observed-adverse-effect level for weekly intraperitoneal or subcutaneous mIMNN-001 administration in mice was 50 µg/animal, equivalent to approximately 6.0 mg/m^2^. Overall, the nonclinical toxicology profile supports the feasibility of repeat administration, with expected local inflammatory effects that were dose-related and reversible rather than associated with sustained systemic toxicity.

The clinical relevance of these local mouse findings should be interpreted in the context of the accumulated human experience. Granulomatous inflammation on peritoneal and serosal surfaces could theoretically lead to transient peritoneal irritation, inflammation, or abdominal pain after intraperitoneal administration. However, the disparity between the murine histopathology and the clinical observations, as discussed in Section 10.2, suggests that the nonclinical response may be influenced by species-specific differences in peritoneal anatomy, innate immune responsiveness, dose exposure, or use of the murine IL-12 construct. Thus, the mouse findings support monitoring for local toxicity but do not establish that persistent granulomatous injury occurs in patients.

## 9. Nonclinical Antitumor Efficacy

### 9.1. Ovarian Cancer Models

As with many immune and targeted therapies in ovarian cancer, monotherapy activity may be constrained by tumor heterogeneity, redundant survival pathways, adaptive immune escape, and a strongly immunosuppressive peritoneal microenvironment. Nevertheless, monotherapy studies are important for defining intrinsic pharmacologic activity, dose response, local cytokine induction, and safety. For IMNN-001, preclinical monotherapy results demonstrated that regional IL-12 expression alone can delay tumor progression and ascites accumulation in ovarian cancer models [58], supporting the biological activity of the platform while also providing a rationale for combinations designed to amplify chemotherapy-induced tumor antigen release and immune priming.

In the ID8 disseminated ovarian cancer model, a tolerated regimen of intravenous carboplatin plus paclitaxel was combined with weekly intraperitoneal murine IMNN-001 (Figure 11). The figure shows the antitumor effects of mIMNN-001, paclitaxel/carboplatin, and their combination in mice with disseminated ID8 ovarian tumors. Untreated animals showed the earliest weight gain, whereas both treatments delayed tumor progression; the combination produced the greatest delay (Figure 11A). Both chemotherapy and IMNN-001 delayed tumor-associated weight gain and improved survival relative to untreated controls, while the combination produced a greater survival benefit than either approach alone (Figure 11B) [58]. Median survival was 42 days for controls, 56 days for chemotherapy, 64 days for mIMNN-001, and 80 days for the combination, which was significantly superior to all other groups (*p* < 0.02). These results supported the concept that repeatable regional IL-12 delivery can be integrated with standard cytotoxic therapy to strengthen antitumor activity.

Toxicology observations from these combination studies were consistent with the intended regional mechanism of IMNN-001. Chemotherapy produced expected dose-dependent hematologic effects, including decreases in white blood cells and platelets, whereas mIMNN-001 did not appear to add systemic toxicity. As described in the nonclinical toxicology GLP study above, weekly intraperitoneal IMNN-001 was associated mainly with mild, dose-related local inflammatory changes in peritoneal tissues that diminished during the 28-day recovery period after the treatment [69]. Together, these findings support the feasibility of combining repeatable intraperitoneal IL-12 gene delivery with standard chemotherapy without substantially increasing systemic toxicity.

Additional combination studies evaluated IMNN-001 with bevacizumab and with bevacizumab plus pegylated liposomal doxorubicin (PLD) in a peritoneally disseminated human SKOV3 ovarian cancer model in athymic nude mice [30]. IMNN-001 or bevacizumab alone each produced antitumor activity, but the combination resulted in a higher proportion of animals without visible tumors at study end. The addition of IMNN-001 to bevacizumab plus PLD produced a marked reduction in tumor burden compared with control and compared with bevacizumab plus PLD alone. Treatments were generally well tolerated, with stable body weights and no meaningful abnormalities in serum chemistry or hematology. These studies further support the concept that local IL-12 gene delivery may complement antiangiogenic and cytotoxic therapies in disseminated ovarian cancer by combining vascular, cytotoxic, and immune-mediated mechanisms of tumor control.

### 9.2. Other Compartmentally Accessible Tumors

Peritoneal carcinomatosis is a clinically important pattern of metastatic spread in gastrointestinal malignancies [70,71,72]. It occurs in a substantial subset of patients with colorectal cancer and is also observed in pancreatic ductal adenocarcinoma, where peritoneal involvement contributes to ascites, bowel obstruction, morbidity, and poor prognosis [72]. These diseases provide an anatomical rationale for evaluating regional intraperitoneal therapy because disseminated implants, ascites-associated cells, and peritoneal surfaces may be exposed without systemic cytokine administration. Accordingly, IMNN-001 has been evaluated preclinically in colorectal and pancreatic peritoneal carcinomatosis models in combination with relevant cytotoxic agents. These findings remain preclinical, and efficacy, safety, dosing, and the possibility of systemic or abscopal immune effects [36] have not been established in patients with gastrointestinal malignancies.

In a disseminated murine CT26 colon carcinoma model, repeated intraperitoneal administration of mIMNN-001 at 50 µg DNA once weekly for 5 weeks, either alone or in combination with 5-fluorouracil at 200 mg/m^2^ once weekly for 2 weeks, improved survival compared with untreated controls [73]. The survival benefit appeared greater with combination therapy than with either treatment alone. Notably, complete tumor rejection occurred in 37.5% of animals treated with mIMNN-001 alone and in 62.5% of animals receiving IMNN-001 plus 5-fluorouracil, compared with 0% in untreated and 5-fluorouracil-only groups. These findings suggest that local IL-12 gene delivery can produce antitumor activity in peritoneally disseminated colorectal cancer and may enhance the effect of fluoropyrimidine chemotherapy.

In a related model of peritoneally disseminated murine PAN-02 pancreatic cancer, intraperitoneal mIMNN-001 administered across a dose range of 25–250 µg DNA produced dose-dependent improvement in overall survival, with the greatest survival benefit observed at 250 µg [73]. Combination treatment with mIMNN-001 plus gemcitabine at 150 mg/kg produced greater survival benefit than either agent alone. These results extend the regional IL-12 delivery concept to pancreatic peritoneal carcinomatosis and support further evaluation of IMNN-001 in combination with chemotherapy where local immune activation may complement cytotoxic tumor control.

Collectively, the colorectal and pancreatic models suggest that IMNN-001 may have utility beyond ovarian cancer, including peritoneal carcinomatosis arising from gastrointestinal malignancies. The common rationale is compartmental disease distribution where intraperitoneal administration can expose disseminated tumor deposits, ascites-associated tumor and immune cells, and peritoneal surfaces to repeatable IL-12 gene delivery while avoiding the high systemic cytokine exposure associated with recombinant IL-12 protein. The apparent compatibility of IMNN-001 with standard chemotherapeutics in these models further supports the potential for combination strategies that integrate cytotoxic tumor debulking with localized immune activation.

#### Compartmental Delivery to Non-Peritoneal Sites

Although the peritoneal carcinomatosis models are most closely aligned with the intended regional application of IMNN-001, evaluation in a glioblastoma model extends the compartmental-delivery concept to a non-peritoneal site accessible by direct local administration. In the orthotopic GL261 glioma model, direct intracranial administration of mIMNN-001 induced local IL-12 expression and increased CD4^+^ and CD8^+^ T-cell infiltration compared with untreated tumors [74]. Both mIMNN-001 and carmustine (BCNU) chemotherapy prolonged survival, while their combination produced the greater benefit, with 100% survival through day 100 [74].

These preclinical findings suggest that the mIMNN-001 platform can support localized IL-12 expression in another anatomically accessible tumor compartment. These preclinical studies are relevant to evaluating the potential of mIMNN-001 in glioblastoma; however, they do not alone establish its clinical applicability in this disease.

## 10. Clinical Rationale and Development in Ovarian Cancer

Management of newly diagnosed advanced epithelial ovarian cancer generally combines cytoreductive surgery, administered either before or after platinum-taxane chemotherapy, with selected use of bevacizumab and maintenance poly (ADP-ribose) polymerase (PARP) inhibitors according to clinical and molecular risk factors [75,76,77,78,79]. Although these approaches have improved disease control, most patients with advanced disease ultimately relapse, and recurrent ovarian cancer frequently becomes progressively less responsive to chemotherapy [75,76,77]. PARP inhibitors provide the greatest benefit in biomarker-defined populations, particularly tumors with breast cancer susceptibility gene (*BRCA*) mutation or homologous recombination deficiency, while benefit is more limited in homologous recombination–proficient disease [78]. Bevacizumab can extend disease control but does not eliminate relapse risk nor does it improve overall survival, and it is associated with class-specific toxicities, including hypertension, proteinuria, and thromboembolic complications [79]. These limitations underscore the continued need for therapies that can improve survival while avoiding excessive systemic toxicity.

Although immunotherapy has delivered durable survival benefits in several cancers, no immunotherapy has demonstrated significant survival effect in ovarian cancer [80,81,82,83,84]. This discrepancy reflects, in part, the immunobiology of ovarian cancer, in which peritoneal tumor deposits and ascites create a compartmentalized and often immunosuppressive microenvironment characterized by T-cell dysfunction, suppressive myeloid populations, regulatory T cells, checkpoint pathway activation, and variable antigenicity [80,81]. Despite their success in several malignancies, immune checkpoint inhibitors have shown limited efficacy in ovarian cancer, with low response rates, and no consistent durable benefit, either as monotherapy or in combination with chemotherapy, antiangiogenic agents, or PARP inhibitors [83,84]. These observations suggest that successful immunotherapy in ovarian cancer may require strategies that actively remodel the peritoneal tumor microenvironment rather than relying solely on checkpoint release.

IMNN-001 was developed to address this challenge through regional, non-viral IL-12 gene delivery. Rather than exposing patients to high circulating concentrations of recombinant IL-12, IMNN-001 is administered intraperitoneally to promote local plasmid uptake and cytokine expression within the peritoneal tumor compartment. This approach is mechanistically attractive because IL-12 can activate natural killer cells and cytotoxic T cells, increase IFN-γ signaling, enhance antigen presentation, and drive a Th1-dominant immune program [65,66,67,68]. By localizing IL-12 production to the disease compartment and allowing repeat administration, IMNN-001 is intended to provide durable immune stimulation at tumor sites while limiting systemic cytokine exposure.

Clinical evaluation of IMNN-001 in ovarian cancer progressed from recurrent, heavily pretreated disease to the newly diagnosed setting, following preclinical evidence that intraperitoneal IMNN-001 delivery could generate local cytokine expression and antitumor activity in peritoneally disseminated ovarian cancer models. Early studies established feasibility, safety, pharmacodynamic localization, and preliminary activity as monotherapy and in combination regimens [30,38,60,61]. Subsequent development has focused on newly diagnosed advanced ovarian cancer [31,62,63], where intraperitoneal delivery can be integrated with neoadjuvant and adjuvant platinum-taxane chemotherapy and where a less treatment-exhausted immune microenvironment may be more amenable to local immune activation.

### 10.1. Recurrent Disease

IMNN-001 was first evaluated in a phase I dose-escalation study in 13 patients with heavily pretreated, platinum-resistant recurrent ovarian cancer [38]. Patients received four weekly intraperitoneal doses after catheter placement across escalating dose cohorts of 0.6, 3, 12, and 24 mg/m^2^. All patients had undergone prior surgery and had received two to six previous chemotherapy regimens. No maximum tolerated dose was reached, and no consistent dose-related safety trend was observed. Most adverse events were mild to moderate, most commonly fever and abdominal pain. Serious adverse events included peritonitis, one event of which was considered possibly treatment related and dose limiting. Overall, this first-in-human study demonstrated that weekly intraperitoneal IMNN-001 could be safely administered in a heavily pretreated population, supporting further evaluation of repeatable regional IL-12 gene delivery.

Pharmacodynamic results from this study provided early clinical support for localized activity. IL-12 plasmid DNA levels were consistently much higher in peritoneal fluid than in blood, and plasmid was detected in only a minority of blood samples. Measurable IL-12 was observed in peritoneal fluid from one patient after the first dose, while IFN-γ increased above baseline in peritoneal fluid in all patients at one or more post-dose time points and was not detected in blood. Higher-dose cohorts had a greater proportion of IFN-γ-positive peritoneal samples than lower-dose cohorts. At five weeks after treatment, 31% of patients had stable disease, with a higher rate in the higher-dose cohorts. These findings suggested that IMNN-001 remained largely confined to the peritoneal cavity and produced biologically active IL-12 signaling with evidence of dose-associated pharmacodynamic activity.

IMNN-001 monotherapy was subsequently evaluated in GOG-0170Q, a multicenter phase II study conducted by Gynecologic Oncology Group in patients with platinum-resistant or persistent epithelial ovarian, fallopian tube, or primary peritoneal cancer [85]. Weekly outpatient intraperitoneal IMNN-001 at 24 mg/m^2^ was administered until disease progression or intolerable toxicity. Toxicities were generally similar to those observed in the phase I monotherapy study, although disease-related events were more frequent in this more advanced population. Among efficacy-evaluable patients, stable disease was observed in 35%, the 6-month progression-free survival rate was 30%, median progression-free survival was 2.9 months, and median overall survival was 9.2 months. Thus, the study indicated limited single-agent activity at 24–36 mg/m^2^ in a heavily pretreated, predominantly platinum-resistant population. This dose range was substantially lower than the 80–100 mg/m^2^ doses currently being evaluated in newly diagnosed disease; therefore, the GOG-0170Q efficacy findings should be interpreted in the context of both the lower dose and the treatment-refractory study population. Nevertheless, the study confirmed the feasibility of repeated outpatient intraperitoneal administration and helped redirect clinical development toward higher-dose combination regimens and the newly diagnosed setting, where earlier intervention, a less treatment-exhausted immune compartment, and chemotherapy-induced tumor-cell death and antigen release may provide a more favorable biological context for IL-12–mediated immune remodeling. Recurrent ovarian cancer remains an important setting for future investigation. Depending on the outcomes of ongoing frontline studies, IMNN-001 could be reevaluated in selected recurrent-disease populations at contemporary doses and in rational combinations with agents such as platinum-based chemotherapy, pegylated liposomal doxorubicin, bevacizumab, PARP inhibitors, immune checkpoint inhibitors, or other therapies capable of enhancing antigen release or reversing immune suppression.

The clinical combination strategy was first explored in a multicenter phase I dose-escalation study of IMNN-001 with carboplatin and docetaxel in patients with platinum-sensitive recurrent ovarian cancer [52]. IMNN-001 was administered intraperitoneally at 12, 18, or 24 mg/m^2^ every 10–11 days for four doses. The regimen was generally manageable, with common adverse events including alopecia, low-grade fever, abdominal pain, and nausea. Three grade 3 events were considered probably related to IMNN-001, including two cases of abdominal pain and one cytokine-release syndrome event with hypotension; however, no deaths, treatment discontinuations, or dose-limiting toxicities occurred. These data supported the feasibility of combining regional IL-12 gene delivery with platinum-taxane chemotherapy.

Among evaluable patients, the objective response rate was 50%, including complete and partial responses, with stable disease in an additional 42%. Ten patients survived more than 12 months and two survived more than 5 years; median progression-free and overall survival were 8.8 and 16.6 months, respectively. Post-treatment peritoneal IFN-γ and TNF-α increased with IMNN-001 dose, although cytokine induction was lower when IMNN-001 was administered concurrently with chemotherapy, likely reflecting corticosteroid premedication for docetaxel. The regimen demonstrated local pharmacodynamic activity and feasibility with chemotherapy, but the reported response and survival findings should be regarded as exploratory.

A second combination approach evaluated IMNN-001 with pegylated liposomal doxorubicin in patients with platinum-resistant ovarian cancer (GOG 9928) [61]. IMNN-001 was administered weekly in combination with PLD given every 28 days across escalating dose cohorts. The most frequent adverse events included leukopenia, anemia, neutropenia, fatigue, constipation, nausea, and abdominal pain. Treatment-related grade 4 events were limited to neutrophil count decreases, and grade 3 events were mainly hematologic or gastrointestinal. No dose-limiting toxicities were reported, supporting the feasibility of repeated intraperitoneal IMNN-001 administration with PLD.

Pharmacodynamic findings again demonstrated preferential cytokine induction in the peritoneal compartment. In evaluable peritoneal fluid samples, IL-12 increased from undetectable baseline levels to measurable concentrations after treatment, while IFN-γ and TNF-α increased substantially and vascular endothelial growth factor (VEGF) declined. Plasma cytokine changes were directionally similar but much smaller, reinforcing the conclusion that IMNN-001 produces predominantly local immune activation after intraperitoneal administration.

Clinical activity in this study was also dose associated. Among response-evaluable patients, the overall objective response rate was 21%, increasing to 28% in the highest-dose cohort, and clinical benefit was 57% overall and 86% in the highest-dose cohort. Median duration of responses ranged from 6.7 to 10.3 months, median progression-free survival was 4.7 months, and median overall survival was 11.1 months. These results supported continued development of IMNN-001 in combination regimens and helped shift the development strategy toward earlier-line ovarian cancer, where immune competence and chemotherapy-induced antigen release may be more favorable for regional IL-12 immunogene therapy.

### 10.2. Newly Diagnosed Disease

The current development of IMNN-001 is centered on newly diagnosed advanced ovarian cancer in combination with neoadjuvant and adjuvant chemotherapy, with additional evaluation alongside antiangiogenic therapy. This setting may be more favorable for immunotherapy than recurrent platinum-resistant disease because patients are less heavily pretreated, may retain broader functional immune competence, and receive platinum-taxane chemotherapy at a time when chemotherapy-induced tumor cell death and antigen release could enhance immune priming. In contrast, recurrent disease is often characterized by cumulative treatment-related immune impairment, resistant tumor clones, T-cell exhaustion, suppressive myeloid and regulatory T-cell populations, and redundant immune escape pathways. Introducing intraperitoneal IL-12 gene delivery earlier in the disease course may therefore provide an opportunity to exploit a more intact immune compartment and remodel the peritoneal tumor microenvironment before it becomes more treatment refractory.

The first newly diagnosed ovarian cancer study of IMNN-001 (OVATION 1) used a standard 3+3 phase I dose-escalation design to evaluate four intraperitoneal doses, 36, 47, 61, and 79 mg/m^2^, in combination with neoadjuvant chemotherapy [62]. IMNN-001 was administered as eight weekly intraperitoneal infusions beginning at Cycle 1 Week 2, together with three 21-day cycles of carboplatin area under the concentration–time curve (AUC) 6 and weekly paclitaxel 80 mg/m^2^. The most common treatment-emergent adverse events considered at least possibly related to treatment included nausea, fatigue, abdominal pain or cramping, anorexia, diarrhea, and vomiting. Grade 4 neutropenia occurred in eight patients and was attributed to chemotherapy. No dose-limiting toxicities occurred. Among 14 response-evaluable patients, 12 had radiologic responses before debulking surgery, including two complete and ten partial responses; nine patients achieved R0 resection, and one patient had a complete pathologic response.

The availability of paired tumor samples before treatment and at debulking surgery provided an opportunity to evaluate treatment-associated changes in the tumor microenvironment. Intraperitoneal IMNN-001 given with neoadjuvant chemotherapy produced dose-related increases in ascitic IL-12 and IFN-γ. IL-12 increased from 3.2-fold at 36 mg/m^2^ to 23-fold at 79 mg/m^2^, while IFN-γ increased from 3.1-fold to 67-fold across the same dose range (Figure 6). In contrast, blood cytokine levels increased only modestly, supporting predominantly local immune activation within the peritoneal compartment.

Translational analyses supported the biological relevance of these cytokine changes. Tumor immunohistochemistry demonstrated reductions in immune-regulatory markers, including Foxp3, PD-1, PD-L1, and IDO1 (Figure 9), together with increased CD8^+^ T-cell infiltration in several patients, as reported in the original publication [54]. Ratios of CD8^+^ cells to immune-regulatory markers increased in most patients, suggesting a shift from an immunosuppressive tumor microenvironment toward a more immune-stimulatory phenotype [54]. These ratios are composite measures of the balance between CD8^+^ T-cell infiltration and immune-regulatory markers, rather than direct measures of CD8^+^ T-cell functional activity. Flow cytometry of peritoneal fluid showed decreased Foxp3^+^ suppressive T cells and a shift from naïve CD8^+^ T cells toward memory effector CD8^+^ cells with greater cytotoxic potential. Collectively, these findings indicate that repeated intraperitoneal IMNN-001 with neoadjuvant chemotherapy can generate localized cytokine induction, reduce suppressive immune-regulatory markers, and promote CD8^+^-dominant immune remodeling in both tumor tissue and the surrounding peritoneal compartment.

Based on these phase I results, IMNN-001 advanced to evaluation at 100 mg/m^2^ with neoadjuvant and adjuvant chemotherapy in a randomized phase I/II study [63]. After a lead-in safety cohort confirmed tolerability of the 100 mg/m^2^ dose, 117 women with International Federation of Gynecology and Obstetrics (FIGO) stage III/IV epithelial ovarian, fallopian tube, or primary peritoneal carcinoma were randomized 1:1 to chemotherapy plus IMNN-001 or chemotherapy alone. Chemotherapy consisted of six 3-week cycles of paclitaxel and carboplatin with interval debulking surgery after Cycle 3; IMNN-001 was delivered intraperitoneally through an implantable catheter for up to 17 doses. Primary objectives were safety and progression-free survival, with overall survival, objective response rate, surgical response, chemotherapy response score, and serologic response as secondary endpoints. Baseline characteristics were generally balanced, although the IMNN-001 arm included more stage IV disease and fewer Eastern Cooperative Oncology Group (ECOG) performance status 0 patients, while PARP inhibitor maintenance was used more often in the control arm. Median chemotherapy exposure was six cycles in both arms, and median IMNN-001 exposure was six doses. Common treatment-related adverse events included neutropenia, nausea, anemia, thrombocytopenia, abdominal pain, fatigue, diarrhea, and vomiting. Abdominal pain was more frequent with IMNN-001 but improved after prophylactic pain medication. Importantly, no cytokine release syndrome, pneumonitis, hepatitis, or serious systemic immune toxicity was reported, supporting the tolerability of repeatable intraperitoneal IL-12 gene delivery in combination with standard chemotherapy.

More broadly, IMNN-001 has been administered with chemotherapy to approximately 100 patients with newly diagnosed advanced ovarian cancer. The overall clinical experience thus far shows no immune-related serious adverse events, despite repeated intraperitoneal dosing and pharmacodynamic evidence of IL-12 and IFN-γ induction (Figure 6C,D). This pattern supports a manageable immune-safety profile and is consistent with predominantly compartmental cytokine activity rather than broad systemic immune activation. Nevertheless, bacterially produced plasmid DNA may theoretically activate innate DNA-sensing pathways through unmethylated CpG motifs and cytosolic DNA sensors, and dedicated assessments of anti-DNA or anti-PPC antibodies, autoimmune responses, and immune sensitization were not uniformly performed. Continued evaluation of these parameters would further characterize the immunologic safety of repeated IMNN-001 administration.

Across the clinical development program, IMNN-001 has been administered to approximately 150 patients, and no persistent treatment-related peritoneal, serosal, or intra-abdominal structural abnormalities corresponding to the granulomatous inflammation observed in mice have been reported. Patients undergo protocol-directed clinical assessments and serial computed tomography imaging for disease monitoring, and no imaging findings indicative of progressive treatment-related local inflammatory injury have been identified. Abdominal pain is more frequent after intraperitoneal IMNN-001 administration and may, at least in part, reflect transient local peritoneal or serosal inflammation; however, the pain generally resolves within approximately 24 h after dosing and has not been associated with persistent abnormalities on serial imaging. Because abdominal pain in patients with advanced ovarian cancer may also reflect malignancy, ascites, catheter procedures, or concomitant therapy, a specific causal relationship cannot be established. The difference between the reversible mouse findings and the accumulated clinical experience further suggests that the granulomatous response may be species specific, although continued surveillance for cumulative local toxicity remains warranted during repeated intraperitoneal administration.

Although the study was not powered for definitive efficacy conclusions, favorable numerical trends were observed in the intent-to-treat population. Median progression-free survival was 14.9 months with IMNN-001 plus chemotherapy compared with 11.9 months with chemotherapy alone, and median overall survival was 46.0 versus 33.0 months, respectively. R0 resection and chemotherapy response score 3 rates were also higher with IMNN-001. Benefits appeared greater among patients receiving PARP inhibitor maintenance and/or with *BRCA*-mutated or homologous recombination-deficient disease. Survival beyond 36 months was 65% with IMNN-001 compared with 50% in the control arm. These results provided clinical support for further evaluation of IMNN-001 as an adjunct to frontline chemotherapy.

At the 100 mg/m^2^ dose, IMNN-001 again increased IL-12, IFN-γ, and TNF-α levels in ascites, whereas plasma cytokine changes remained modest (Figure 6) [31]. The magnitude of cytokine induction suggested a plateau relative to the dose–response observed in the phase I newly diagnosed study. Tumor microenvironment analyses showed immune changes broadly consistent with those observed previously, including shifts in immunostimulatory and immune-regulatory markers and increased immunostimulatory-to-immunoregulatory cell ratios. These findings further support the central concept that IMNN-001 can provide repeated local cytokine induction and immune remodeling within the peritoneal compartment without producing large systemic cytokine excursions.

### 10.3. Continuing Investigations

Two ongoing randomized multicenter studies are evaluating IMNN-001 in newly diagnosed advanced ovarian cancer, building on the clinical activity and pharmacodynamic localization observed in the phase I and phase II OVATION studies [31,62,63]. A phase I/II minimal residual disease study is evaluating standard platinum-taxane chemotherapy plus bevacizumab, with or without intraperitoneal IMNN-001 at 80 mg/m^2^, in an open-label randomized design. The protocol includes neoadjuvant chemotherapy, interval debulking surgery, adjuvant chemotherapy, bevacizumab during chemotherapy and maintenance, and weekly IMNN-001 beginning during Cycle 1 with continued every-3-week dosing during bevacizumab maintenance for eligible *BRCA*-wild-type, homologous recombination-proficient patients. The primary endpoint is minimal residual disease assessed by second-look laparoscopy, with progression-free and overall survival as secondary endpoints. In parallel, the confirmatory phase III OVATION-3 trial is evaluating intraperitoneal IMNN-001 at 100 mg/m^2^ plus standard neoadjuvant and adjuvant chemotherapy versus chemotherapy alone. OVATION-3 will enroll an all-comer’s population of patients with both homologous recombination-deficient disease and homologous recombination-proficient cohort; eligible patients who respond to first-line platinum-based chemotherapy will proceed to PARP inhibitor maintenance according to applicable guidelines and prescribing information. The primary endpoint is overall survival, with secondary endpoints including chemotherapy response score, surgical response score at interval debulking surgery, time to second-line treatment or death, and objective response rate.

## 11. Chemistry, Manufacturing, and Controls (CMC), Stability, and Clinical Administration Considerations

### 11.1. Clinical-Grade IMNN-001 Manufacturing

The clinical manufacturing strategy for IMNN-001 encompasses the final lyophilized drug product, the IL-12 expression plasmid, and the PPC facilitating agent. Each component has advanced from bench-scale development to reproducible clinical-scale manufacture, with process controls established for plasmid production and purification, PPC synthesis and isolation, nanoparticle formulation, sterile filtration, vial filling, and lyophilization. The unit operations used for all three elements are conventional, scalable, and adaptable to larger-scale commercial current Good Manufacturing Practice (cGMP) production as clinical demand increases.

The hIL-12 plasmid DNA drug substance is produced by fed-batch fermentation of an *E. coli* production strain. A seed culture grown to exponential phase is used to inoculate a stirred-tank bioreactor containing microbial base medium. After depletion of the glycerol carbon source, a feed medium containing yeast extract, glycerol, and other supplements is added at a controlled rate. At a predetermined time, plasmid production is induced. After cooling, the bacterial paste is harvested by centrifugation, resuspended in buffer, and subjected to continuous alkaline lysis, followed by ultrafiltration and chromatographic polishing. Final buffer exchange and bioburden-reduction filtration yield the drug substance, which is stored at −80 °C until use. The clinical lots manufactured under cGMP guidelines passed the quality control specifications using qualified and validated tests for sterility, endotoxin content, residual solvent, identity, sequencing, DNA concentration, purity, homogeneity, pH, genomic host *E. coli* DNA, RNA and protein.

PPC is produced by the covalent attachment of activated PEG and cholesterol to a branched polyethyleneimine (PEI) core. The crude product is dried, acidified with aqueous HCl, and concentrated. The viscous solution is added to acetone, which serves to precipitate the desired product while solubilizing impurities. The resulting polycationic copolymer is collected by filtration, dried, and stored as a solid product at −20 °C until use. Several cGMP lots of PPC were synthesized and released after GLP quality testing by validated and qualified assays for visual inspection, identity, elemental and molecular composition, moisture content, residual solvents, endotoxin and sterility. Additional routine QC testing of PPC included nanoparticles of defined size and promoted gene transfer in cell culture.

The IMNN-001 drug product is a formulation of discrete nanoparticles. The hIL-12 plasmid DNA and PPC are suspended separately in 3% lactose solutions and sterile-filtered. Lactose serves as a cryoprotectant during freezing and as a cytoprotectant and stabilizing matrix during drying and subsequent storage [86]. Streams of each solution are combined and passed through a helical static mixer at a controlled rate to promote the electrostatic complexation of cationic PPC and anionic plasmid DNA. The lactose-stabilized solution is then injected into glass vials and lyophilized to produce the final product cake, stored at −20 °C until use. Immediately prior to administration, the dried cake is resuspended in water for injection (WFI) and placed into an intravenous (IV) bag where it is infused through a catheter placed in the intraperitoneal space. All clinical lots manufactured under cGMP guidelines passed the quality control specifications for product sterility, endotoxin content, DNA concentration, moisture content, pH, osmolality, particle size and potency.

### 11.2. Stability Studies

The long-term stability profile of IMNN-001 has been characterized by using GMP lots representative of clinical-study material. Stability studies evaluated storage at −80 °C ± 5 °C, −20 °C ± 5 °C, 5 °C ± 2 °C, and 25 °C ± 2 °C/60% relative humidity ±5%, with testing through 24 to 36 months. Across these studies, IMNN-001 remained within specification for appearance, DNA concentration, pH, moisture content, sterility, and other key release attributes under frozen storage. Particle size and osmolality, assessed using non-qualified methods, were also maintained. Biological activity in a non-qualified in vitro IL-12 expression assay remained stable under frozen storage but declined over time at 25 °C and at 5 °C. These findings support frozen storage at or below −20 °C to preserve biological activity and maintain product performance for clinical administration. The data from 3-year stability study at −20 °C (5% humidity), the current storage temperature for clinical product IMNN-001, is shown in Table 1.

Post-reconstitution stability was also evaluated to support clinical handling and preparation. IMNN-001 was reconstituted with water for injection to a DNA concentration of 0.5 mg/mL in isotonic lactose and stored at 4 °C. At 60 and 90 days, DNA concentration, particle size, osmolality, and IL-12 gene-expression activity in transfected COS-1 cells were compared with those of the lyophilized product stored in sealed vials at −80 °C. Reconstituted IMNN-001 remained stable at 4 °C for at least 60 days, with no significant changes in DNA concentration, particle size, osmolality, or gene-expression activity relative to the lyophilized comparator.

Together with catheter-compatibility findings as described below, these stability data support the logistical practicality of IMNN-001 as a repeat-administered intraperitoneal therapy and reinforce the feasibility of maintaining local, durable, and repeatable IL-12 delivery in clinical studies.

### 11.3. Catheter Compatibility

Repeat intraperitoneal administration of IMNN-001 requires a delivery system that preserves formulation integrity during catheter-based infusion. In clinical studies, IMNN-001 has been administered through subcutaneously implantable intraperitoneal silicone catheters, including PORT-A-CATH, Quinton Curl, and Bard devices. Compatibility testing under simulated clinical-use condition was conducted with PORT-A-CATH and Quinton Curl silicone catheters demonstrated that IMNN-001 was compatible with both test devices. Recovered material showed no meaningful changes in particle size, osmolality, gene-transfer activity, or DNA recovery, indicating that the tested catheter materials did not adversely affect IMNN-001 potency or formulation stability. These findings support the practical feasibility of repeat intraperitoneal dosing, an essential feature of the local, durable, and repeatable IL-12 delivery strategy.

## 12. Additional IL-12 Delivery Approaches

The limitations of systemic recombinant IL-12 have stimulated development of multiple delivery strategies intended to improve therapeutic index by controlling where, when, and for how long IL-12 is expressed (Table 2). These approaches include tumor-targeted immunocytokines and fusion proteins, conventional and self-replicating mRNA formulations, physical delivery of plasmid DNA, extracellular-vesicle and exosome systems, replication-deficient viral vectors, IL-12–armed oncolytic viruses, and regional non-viral plasmid systems [87,88,89,90,91,92,93,94,95,96,97,98,99,100,101,102,103,104]. Although these modalities differ in composition, administration route, expression kinetics, clinical maturity, and capacity for repeat dosing, they share the objective of preserving IL-12 immune activity while reducing the systemic cytokine peaks and downstream inflammatory toxicities that constrained recombinant protein programs.

Tumor-targeted IL-12 immunocytokines and fusion proteins seek to enrich cytokine activity within tumors through antibody binding, extracellular-matrix association, or engineered retention while extending circulating half-life [87,88,89,90]. NHS-IL12/M9241, for example, targets exposed DNA–histone complexes in necrotic tumor regions. These constructs can improve tumor exposure relative to unmodified recombinant IL-12, but they remain systemically administered; targeting may be incomplete, peripheral immune activation can persist, and the prolonged exposure created by Fc engineering may complicate rapid control of cytokine-related toxicity [88,89,90].

mRNA delivery has emerged as a particularly active IL-12 strategy because cytoplasmic translation avoids the requirement for nuclear entry and does not introduce a DNA template with theoretical integration risk. The first clinical example, MEDI1191, uses an ionizable lipid nanoparticle optimized for intratumoral delivery of mRNA. In a phase I study with durvalumab, injected lesions showed IL-12/IFN-γ pharmacodynamic activity, increased inflammatory gene signatures, and greater immune-cell infiltration, establishing clinical proof of mechanism for local IL-12 mRNA expression [91]. However, intratumoral administration requires lesions that are accessible directly or through image guidance; heterogeneous injection coverage, variable lesion size and distribution, and the need for repeated procedures may limit treatment of diffuse or anatomically difficult disease.

Recent preclinical studies broaden the mRNA delivery concept beyond direct lesion injection. Intravenous administration of an IL-12–encoding self-replicating RNA lipid nanoparticle (JCXH-211) generated tumor IL-12 expression, remodeled myeloid and lymphoid compartments, enhanced checkpoint blockade, and produced antitumor activity in multiple mouse models without evident systemic toxicity in tumor-bearing mice or non-human primates [92]. Self-replicating RNA may prolong protein production at a lower RNA dose, but the duration and anatomical distribution of expression require careful control because systemic delivery could amplify off-target cytokine production. Inhaled extracellular vesicles carrying IL-12 mRNA preferentially delivered the transcript to lung tumors, activated innate and adaptive immunity, and generated systemic antitumor memory in preclinical lung-cancer models [93]. A pancreas-targeted lipid nanoparticle similarly enabled relatively non-invasive IL-12 mRNA delivery in orthotopic pancreatic ductal adenocarcinoma [87]. These organ-directed approaches illustrate how route and carrier tropism may substitute for direct tumor injection, although reproducibility of tissue selectivity, interpatient variability, payload distribution to normal tissue, and scalable manufacture remain unresolved translational issues.

Combination mRNA nanoparticles are also being designed to address adaptive immune suppression. Local co-delivery of IL-12 mRNA with an indoximod prodrug increased CD8^+^ effector function while countering regulatory T-cell responses in preclinical tumor models [94]. More recent systems co-formulated IL-12 mRNA with doxorubicin in polymeric nanoparticles or paired IL-12 mRNA with PD-L1 siRNA in peptide-modified lipid nanoparticles for glioblastoma [95,96]. These multifunctional formulations may synchronize cytokine expression with chemotherapy or checkpoint-pathway inhibition, but they introduce added complexity in cargo ratio, release kinetics, potency testing, toxicology attribution, and manufacturing control. Importantly, most remain preclinical; consequently, their comparative safety, repeat-dose feasibility, and clinical benefit relative to single-cargo IL-12 platforms are not yet established.

Extracellular vesicles (EVs), including exosomes, are biologically derived alternatives to synthetic nanoparticles. Their lipid bilayers protect nucleic acids and may facilitate cellular uptake and tissue-selective distribution. IL-12 EV strategies include surface display of active cytokine and encapsulation of IL-12 mRNA [93,97]. Surface display can retain IL-12 locally without recipient-cell transfection, whereas mRNA loading enables transient intracellular production after uptake; the two formats require distinct pharmacologic, potency, and manufacturing controls [93,97].

In exoIL-12, functional IL-12 is fused to the exosomal membrane protein PTGFRN and displayed on the vesicle surface. Intratumoral administration in preclinical models produced approximately 10-fold greater tumor exposure than recombinant IL-12, sustained local IFN-γ induction for up to 48 h, increased tumor-specific CD8^+^ T cells, and generated immune memory [97]. Surface anchoring may reduce cytokine diffusion, but treatment still requires an accessible tumor or another localized route, and systemic leakage, dose relationships, and comparative clinical efficacy remain undefined.

Inhaled IL-12 mRNA exosomes extend EV delivery to multifocal pulmonary tumors without lesion-by-lesion injection. In preclinical models, electroporation-loaded, human embryonic kidney cell-derived exosomes preferentially entered lung tumors, induced local IL-12 and IFN-γ, enhanced antigen presentation and cytotoxic immunity, and protected against tumor rechallenge [93]. Activity with checkpoint inhibition and preliminary tolerability in mice and non-human primates supported further investigation [93]. However, pulmonary deposition may vary with airway anatomy, tumor location, device performance, and lung function, and other organs would require alternative routes or targeting.

EV products pose challenges in controlling vesicle identity, composition, cargo loading, surface IL-12 density, residual producer-cell material, sterility, potency, and batch consistency [98,99]. Electroporation may cause variable mRNA loading or aggregation, while surface-engineered products must retain cytokine orientation and bioactivity [93,97]. Scale-up, purification, stability, biodistribution, immunogenicity, and repeat-dose safety remain incompletely defined, and EVs are neither necessarily non-immunogenic nor tumor specific [98,99]. Unlike EV systems, IMNN-001 uses a chemically defined polymer for compartment-wide plasmid delivery; no head-to-head studies establish superiority in efficacy, safety, duration, manufacture, or clinical practicality.

Beyond IL-12–specific mRNA and EV platforms, recent stimulus-responsive chemo-immunotherapy systems illustrate complementary design principles that may inform future delivery strategies. A tumor-cell-derived biomimetic microparticle co-delivering syrosingopine and the acid-activated doxorubicin prodrug Dox-EMCH disrupts intracellular and extracellular pH homeostasis, promotes immunogenic cell death, and reverses features of an acidic immunosuppressive microenvironment [100]. Similarly, tumor-acidity-responsive polymeric nanoparticles co-delivering norcantharidin and a platinum compound combine environmentally triggered release with inhibition of tumor-cell defense pathways, including PD-L1 upregulation [101]. These preclinical systems demonstrate potential advantages of tumor targeting, synchronized combination-payload delivery, and microenvironment-responsive release. However, unlike the IL-12 EV approaches discussed above or IMNN-001, they deliver small-molecule chemo-immunotherapy payloads rather than a cytokine or cytokine-encoding nucleic acid, were evaluated in different tumor models, and do not address repeated regional gene expression in disseminated ovarian cancer. Accordingly, they provide useful conceptual comparators, but direct quantitative comparison with IMNN-001 would not be scientifically appropriate.

Oncolytic viruses combine selective tumor-cell infection and lysis with local expression of IL-12, potentially coupling antigen release, innate inflammation, and cytokine-driven adaptive immunity. Platforms based on herpes simplex virus [102,103], adenovirus [102], vaccinia virus [102,104], and other backbones have been evaluated preclinically, and early clinical studies of IL-12–armed oncolytic viruses have begun to assess intratumoral safety and activity. Their principal challenges include variable tumor permissiveness, antiviral immunity, inflammatory toxicity, genetic stability of the inserted payload, biosafety and shedding controls, complex manufacture, and the need for direct tumor access in many programs.

IMNN-001 differs from these approaches by combining regional administration with a non-viral plasmid/PPC delivery system and is among the most clinically advanced IL-12 delivery strategies in ovarian cancer, having progressed through multiple clinical studies into an ongoing phase III trial. For ovarian cancer specifically, the most directly relevant precedent is regional IL-12 exposure to the peritoneal cavity. Historical intraperitoneal recombinant IL-12 studies established the feasibility and risks of cytokine delivery to this compartment, while IMNN-001 represents a more advanced ovarian cancer–focused strategy designed to convert peritoneal IL-12 exposure from repeated protein administration into repeatable local gene expression. Other clinical-stage IL-12 platforms provide important proof of principle in solid tumors, but they are not yet established as ovarian cancer-specific delivery strategies. Accordingly, IMNN-001 is best viewed as a compartmentally targeted IL-12 gene therapy whose differentiation is strongest in diseases where the tumor burden is anatomically accessible to regional dosing.

## 13. Limitations, Future Directions and Conclusions

IL-12 remains one of the most biologically compelling cytokines for cancer immunotherapy, but its clinical use has historically been constrained by systemic inflammatory toxicity and inadequate tumor-local exposure. IMNN-001 addresses these limitations through a compartmentally targeted, non-viral TheraPlas platform designed to produce IL-12 locally after intraperitoneal administration, sustain biologically active expression over a clinically meaningful interval, and permit repeat dosing to reinforce immune activation over time. This delivery profile distinguishes IMNN-001 from systemic recombinant IL-12, tumor-targeted immunocytokines, intratumoral mRNA approaches, and viral or oncolytic IL-12 platforms.

Across preclinical and clinical studies, IMNN-001 has produced preferential cytokine induction in the peritoneal compartment, including IL-12 and downstream IFN-γ, with smaller systemic cytokine changes. Associated pharmacodynamic findings include increased cytotoxic-effector activity, higher immunostimulatory-to-immune-regulatory marker ratios, and reduced suppressive immune signals in tumor and ascites samples. Clinical administration has generally been feasible, but important risks and limitations include abdominal pain, catheter-related complications, one reported cytokine-release syndrome event in a recurrent-disease combination study, and reversible local peritoneal inflammation in nonclinical studies. The frequency of abdominal pain is manageable with the use of prophylactic pain medication in recent studies. The existing data supports continued evaluation; the ongoing randomized phase III OVATION-3 trial will determine whether adding IMNN-001 to standard chemotherapy improves overall survival and will further characterize its safety profile in newly diagnosed advanced ovarian cancer. Biodistribution, toxicology, stability, and catheter compatibility further support of repeated regional administration, although they do not establish comparative superiority or definitive long-term safety.

Future development will clarify how best to integrate IMNN-001 into contemporary ovarian cancer treatment, including its use with platinum-taxane chemotherapy, bevacizumab, and PARP inhibitor maintenance according to molecular risk groups, and identify patient populations most likely to benefit from the treatment and the potential role of biomarkers such as homologous recombination status and *BRCA* mutation status. Ongoing randomized studies, including OVATION-3 and minimal residual disease–focused investigations, will be important for determining whether the local immune remodeling observed to date translates into durable improvements in survival and disease control.

Additional product-characterization studies will be informative, directly assessing PPC tissue distribution, degradation kinetics, metabolite formation, and clearance pathways. Such studies will help distinguish the in vivo fate of the carrier from that of the plasmid cargo and determine the relative contributions of renal, hepatobiliary, and cellular processing to elimination of the intact conjugate and its degradation products.

Taken together, these remaining mechanistic and carrier-disposition questions provide the context for assessing the continued use of PPC. Its rationale is supported by defined plasmid-condensation and protection properties, regional intraperitoneal pharmacodynamics, scalable manufacturing, stability, catheter compatibility, repeated administration, and accumulated ovarian-cancer clinical experience. The antibiotic-resistance marker in the clinical plasmid is also relevant to regulatory and biosafety assessment. Current cGMP purification, release testing, intraperitoneal administration, and progressive plasmid clearance provide risk-mitigation measures but do not demonstrate that horizontal transfer is impossible. Future-generation IMNN-001 constructs could evaluate antibiotic-free selection or reduced bacterial-backbone designs to eliminate the resistance marker and potentially reduce backbone-associated innate immune stimulation.

Several additional areas merit consideration as IMNN-001 advances. Further physicochemical and mechanistic characterization should define nanoparticle morphology, cellular uptake, intracellular trafficking, endosomal escape, PPC–DNA decomplexation, carrier degradation, metabolite formation, and clearance. Genomic-safety studies capable of distinguishing persistent episomal plasmid from rare integrated sequences, including targeted evaluation of reproductive tissues, would further clarify theoretical insertional and germline risks. Although clinical experience in approximately 150 newly diagnosed ovarian cancer patients supports manageable immune safety, longitudinal assessment of anti-DNA and anti-PPC responses, innate immune activation, immune sensitization, cumulative local inflammation, and pharmacodynamic consistency after repeated dosing will be useful.

In addition to addressing product characterization and safety profiles, ongoing clinical trial will clarify the most effective role for IMNN-001 in treating ovarian cancer. Potential areas for further clinical development include dose and schedule optimization, standardization of pharmacodynamic measurements in ascites and plasma, and development of predictive biomarkers of cytokine induction, immune remodeling, and clinical benefit. The ongoing OVATION-3 study incorporates prospective assessment of *BRCA* mutation and homologous-recombination status, while baseline immune-cell composition and suppressive-marker profiles remain additional candidates for prospective evaluation to further define patients most likely to benefit. Combination strategies with immune checkpoint inhibitors may be investigated and optimized according to biological rationale, sequence, and molecular subgroup rather than assumed to be uniformly additive. Most importantly, the favorable efficacy signals observed to date are being evaluated in the ongoing randomized, statistically powered phase III OVATION-3 trial, with overall survival as the primary endpoint.

Beyond ovarian cancer, the broader translational opportunity for IMNN-001 and related non-viral IL-12 gene delivery platforms may lie in anatomically accessible tumor compartments where regional administration can concentrate cytokine activity at disease sites while limiting systemic exposure. Peritoneal carcinomatosis of gastrointestinal origin and locally accessible brain tumors provide preclinical examples of this principle. More broadly, IMNN-001 illustrates a delivery-based solution to a long-standing cytokine immunotherapy challenge: preserving the antitumor potency of IL-12 by controlling its spatial distribution, duration of expression, and repeatability of administration.

## Figures and Tables

**Figure 1:**
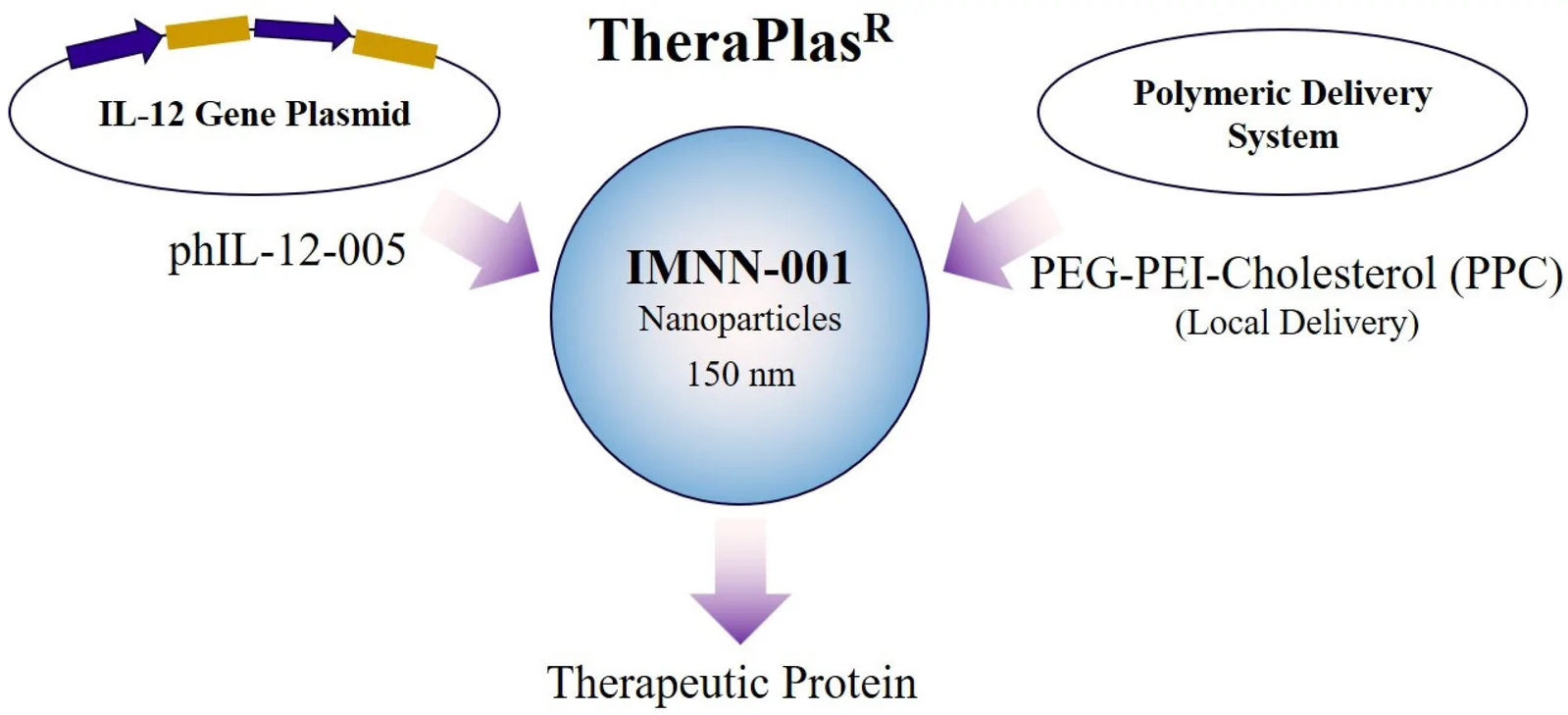
TheraPlas platform for gene delivery of therapeutic proteins. A TheraPlas product consists of a gene-expression plasmid, such as an IL-12 plasmid (phIL-12-005), and a plasmid delivery system, such as PPC, which are combined under controlled formulation conditions to form therapeutic nanoparticles, exemplified by IMNN-001. The plasmid encodes the IL-12 subunit genes *p35* and *p40* (golden-yellow dashed arcs), each driven by a separate cytomegalovirus (CMV) promoter (dark blue arrows).

**Figure 2:**
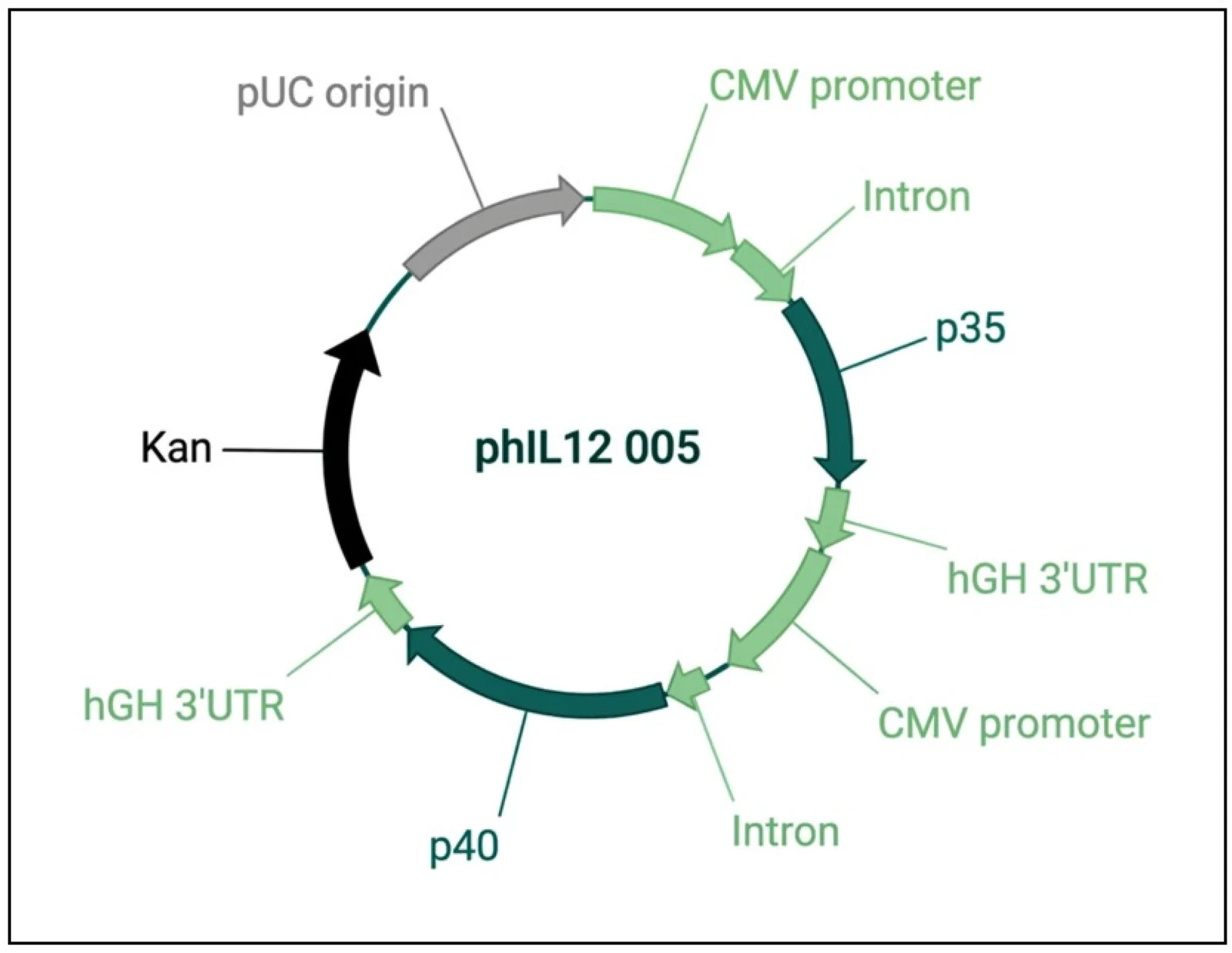
IMNN-001 IL-12 gene expression system. The expression plasmid phIL-12-005 contains cDNA sequences encoding both IL-12 subunits, *p35* and *p40*, each driven by an independent cytomegalovirus immediate-early promoter. The plasmid also includes intronic sequences, 3′ untranslated regions, polyadenylation elements, and a kanamycin-resistance gene within the plasmid backbone.

**Figure 3:**
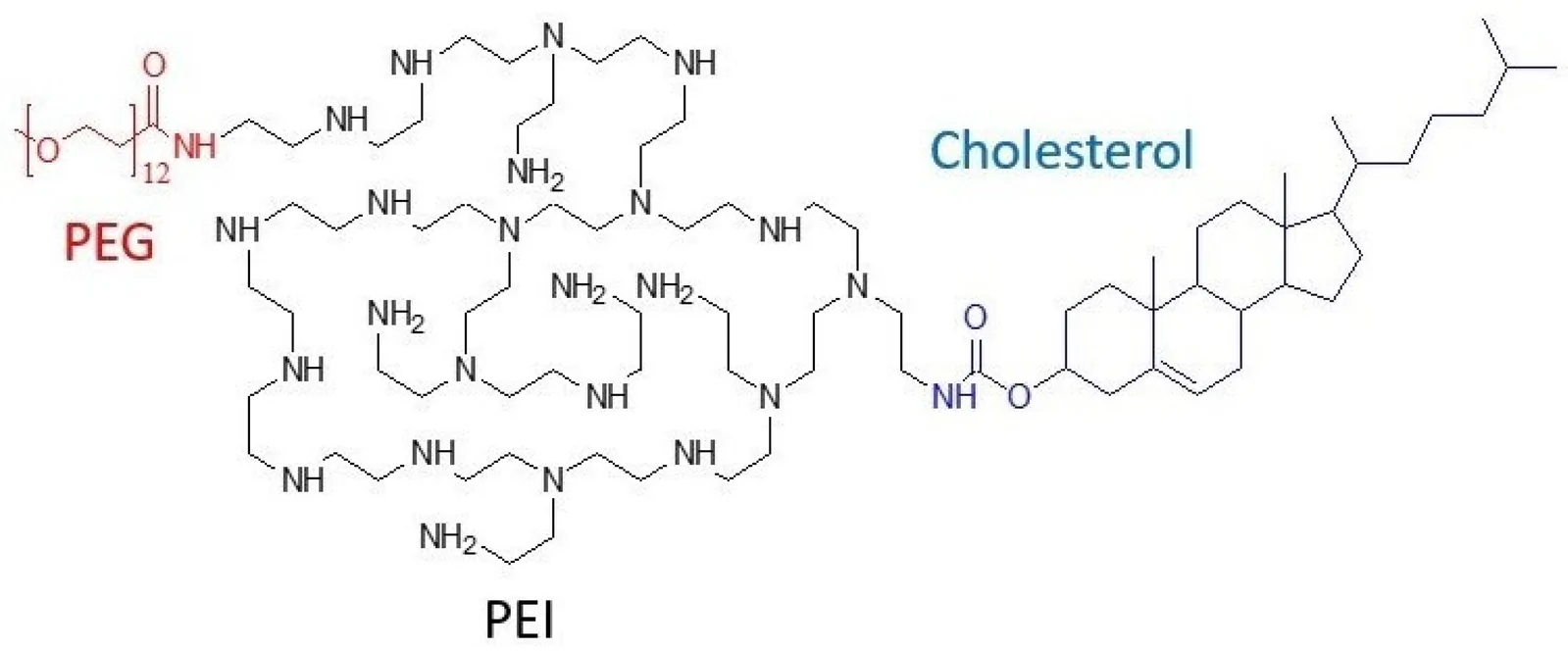
Gene delivery polymer, PPC. Chemical structure comprising a PEI backbone covalently conjugated to PEG and cholesterol.

**Figure 4:**
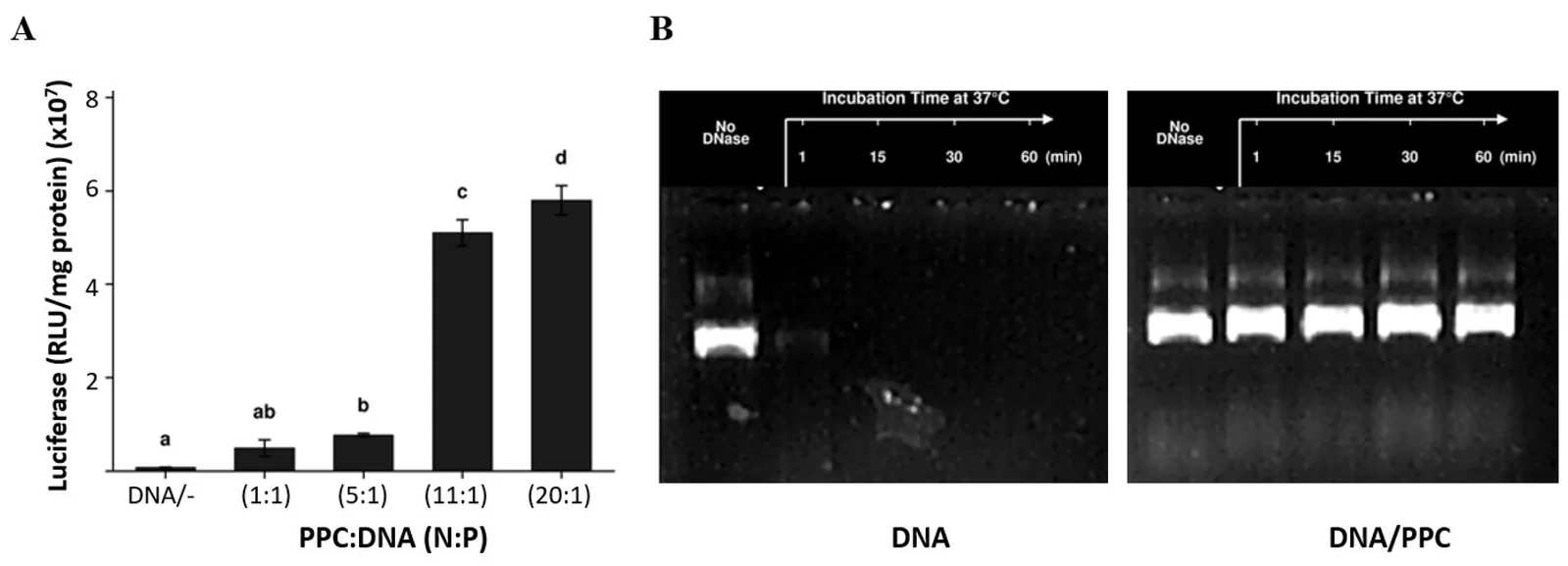
Optimization of PPC-mediated transfection and plasmid protection. (**A**) Transfection efficiency in COS-1 cells transfected with 4.0 µg luciferase expression plasmid complexed with PPC at varying N:P ratios. Values were compared by one-way analysis of variance (ANOVA) with Duncan’s multiple-comparison method; statistical significance (*p* < 0.05) is indicated by lowercase letters. (**B**) Nuclease protection assay demonstrating that complexation with PPC protects plasmid DNA from DNAse-mediated degradation (right panel). Following DNAse incubation, the DNA was released from the nanoparticles by decomplexation with Dextran sulfate. Unprotected plasmid DNA was degraded under the same conditions (left panel). Reproduced with permission from [37] Elsevier Limited (2005); permission conveyed through Copyright Clearance Center, Inc.

**Figure 5:**
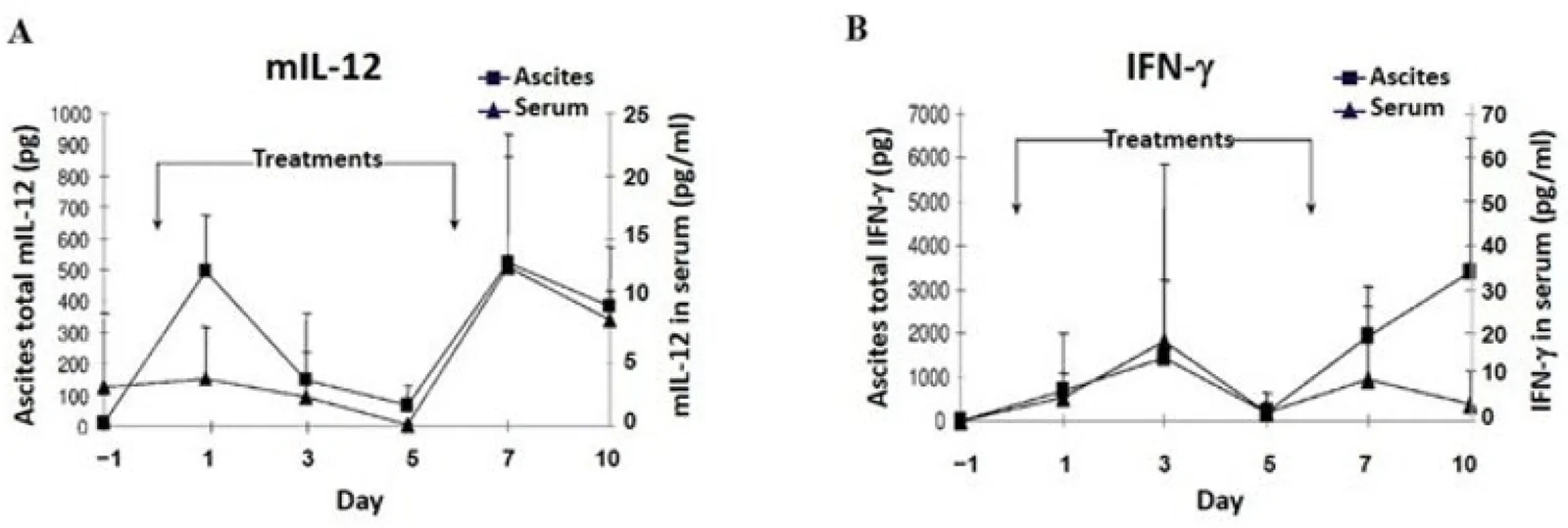
Local and repeatable gene delivery of IL-12 in a mouse model of ovarian cancer. (**A**) IL-12 levels and (**B**) IFN-γ levels in ascites (pg total, left scale) and serum (pg/mL, right scale) following repeated intraperitoneal mIMNN-001 treatment in mice with disseminated ID8 ovarian tumors.. Arrows indicate pmIL-12/PPC administration on days 0 and 6. On a pg/mL basis, assuming a total blood volume of 1.5–2 mL in a 20–25 g mouse and an ascites volume of 5–10 mL, the cytokine levels in ascites were several-fold higher than in serum. Values are mean ± standard deviation (SD), with *n* = 6 for each time point. Reproduced with permission from [58] John Wiley and Sons (2009); permission conveyed through Copyright Clearance Center, Inc.

**Figure 6:**
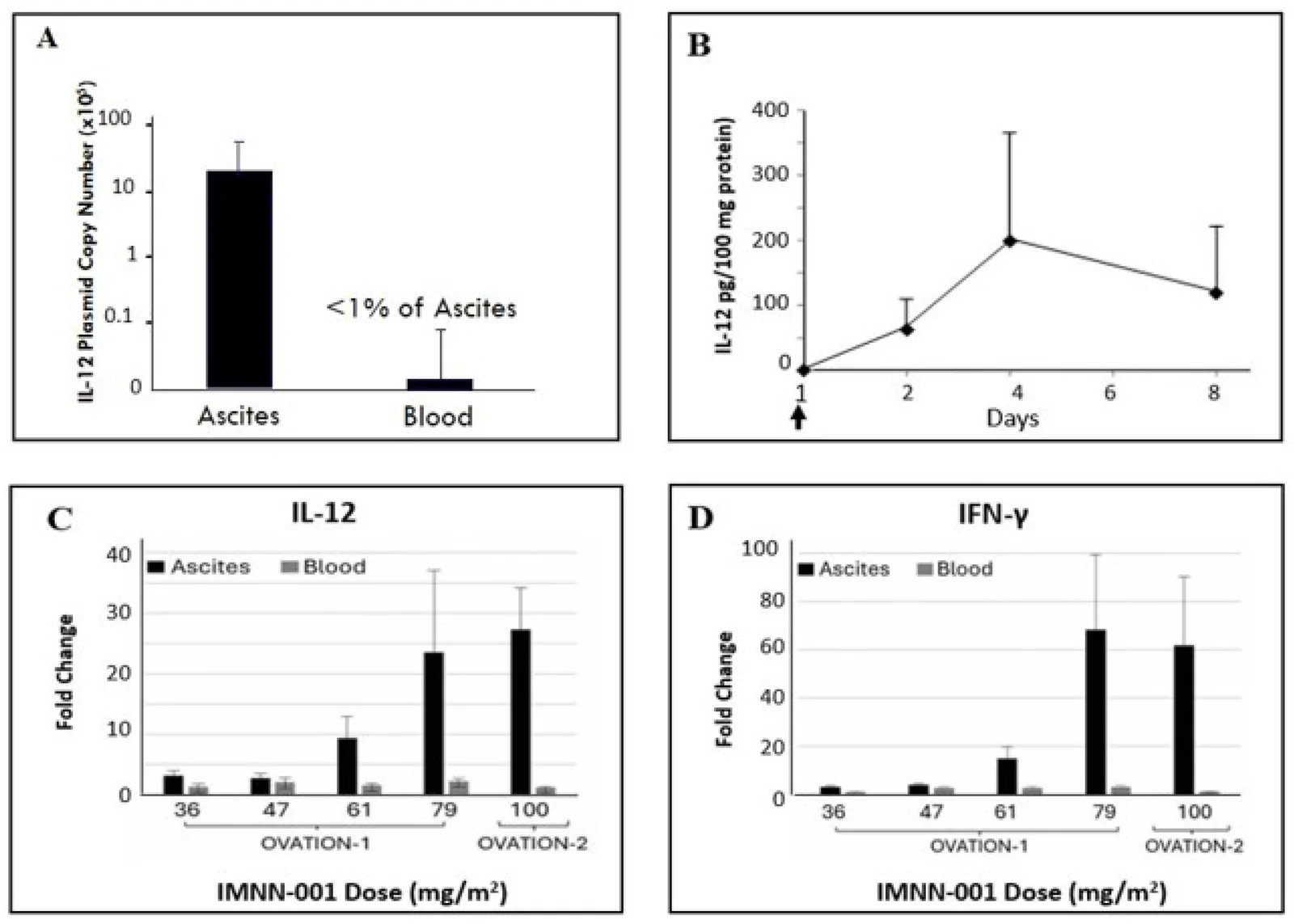
Compartment-selective plasmid distribution and cytokine induction following intraperitoneal IMNN-001 treatment in ovarian cancer clinical studies. (**A**) Plasmid distribution in ascites and blood following intraperitoneal IMNN-001 treatment in patients with recurrent ovarian cancer. Adapted with permission from [38] Springer Nature (2009); permission conveyed through Copyright Clearance Center, Inc. (**B**) Changes in IL-12 levels in ascites at various timepoints after intraperitoneal IMNN-001 administration (arrow). Reproduced from Thaker et al. [61]. (**C**,**D**) Dose-dependent ascitic IL-12 and IFN-γ induction in patients with newly diagnosed advanced ovarian cancer treated with intraperitoneal IMNN-001 and neoadjuvant chemotherapy. Reproduced from Thaker et al. [31].

**Figure 7:**
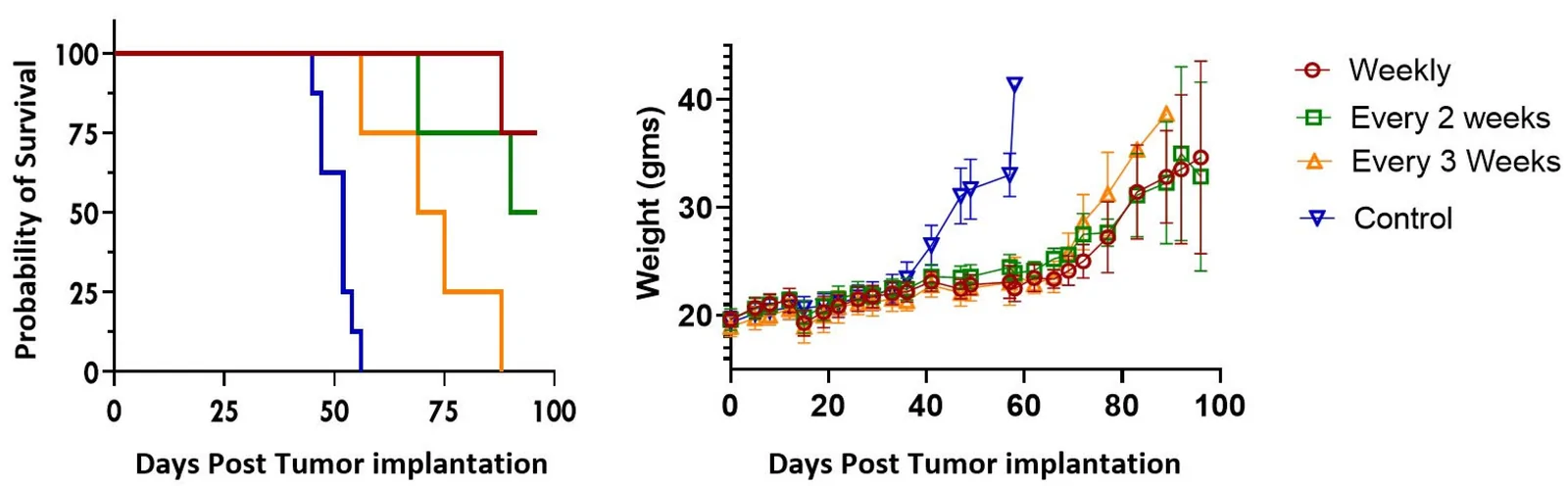
Treatment frequency and survival in disseminated mouse ID8 ovarian tumor model. Effect of intraperitoneal IMNN-001 dosing interval on tumor burden, measured by body weight (**right panel**) and survival (**left panel**) in mice bearing peritoneally disseminated ID8 ovarian tumors. Reproduced from Boyer et al. [64].

**Figure 8:**
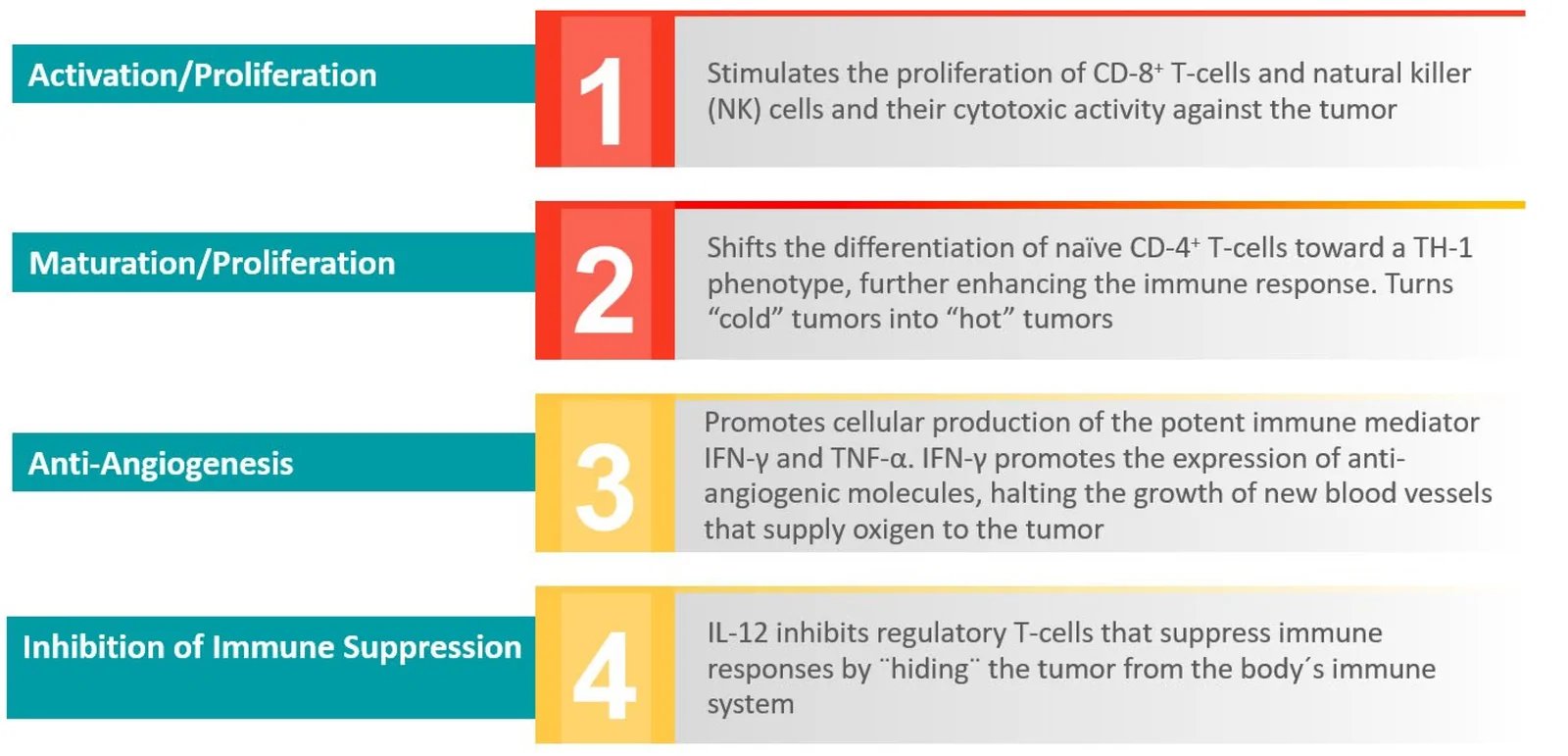
IL-12-mediated immune activation and remodeling of the tumor microenvironment. IL-12 promotes coordinated innate and adaptive antitumor immunity by activating natural killer cells and cytotoxic CD8^+^ T cells, enhancing IFN-γ production, supporting Th1 polarization, and improving dendritic-cell maturation and antigen presentation. Through these effects, IL-12 can shift an immunosuppressive tumor microenvironment toward a more immune-stimulatory state and may also contribute to indirect antiangiogenic activity.

**Figure 9:**
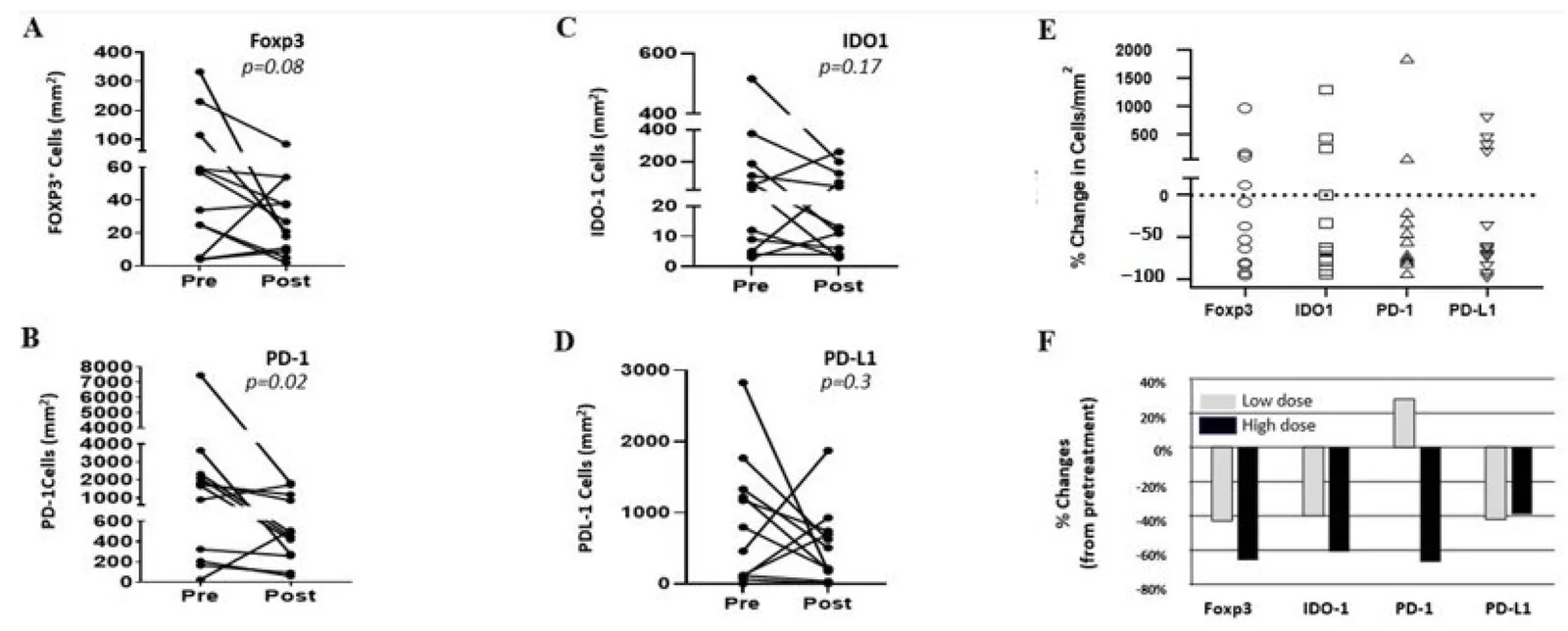
IMNN-001–associated modulation of immunosuppressive markers in the ovarian tumor microenvironment. Treatment-related changes in tumor expression of Foxp3, PD-1, IDO1, and PD-L1 are shown before treatment (pre) and at interval debulking surgery (post) in individual patients. (**A**–**D**) Paired pre- and post-treatment levels of Foxp3, PD-1, IDO1, and PD-L1. (**E**) Percent change from baseline for each marker. (**F**) Percent change by dose group, defined as low dose, 36 and 47 mg/m^2^ (*n* = 4), and high dose, 61 and 79 mg/m^2^ (*n* = 8). Paired pre- and post-treatment values were compared using the Wilcoxon matched-pairs signed-rank test. Reproduced from Thaker et al. [62].

**Figure 10:**
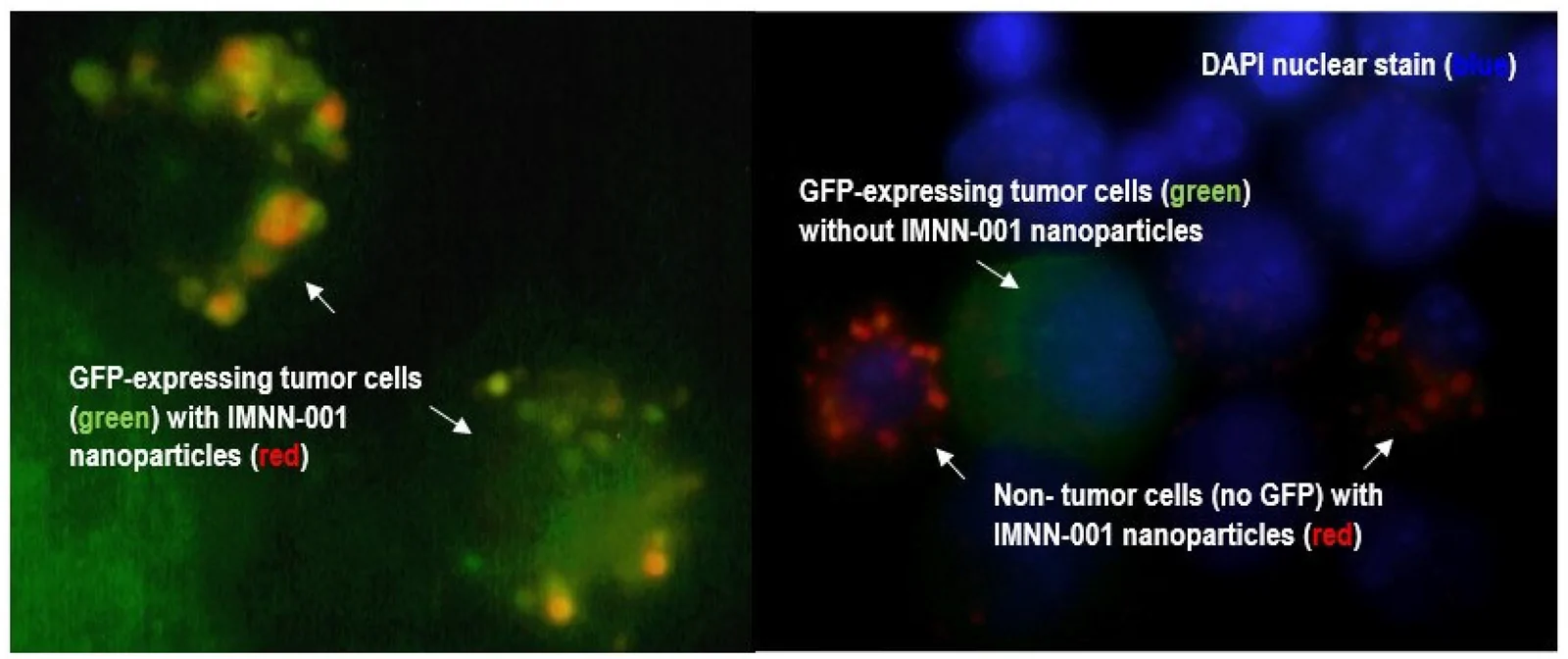
Intracellular distribution of fluorescently labeled IMNN-001 nanoparticles in ascites of disseminated mouse ID8 ovarian tumor model. Panels show PPC localization (red), GFP-expressing ID8 tumor cells (green), and 4′,6-diamidino-2-phenylindole (DAPI) nuclear staining (blue) 24 h IMNN-001 administration. Reproduced with permission from [58] John Wiley and Sons (2009); permission conveyed through Copyright Clearance Center, Inc.

**Figure 11:**
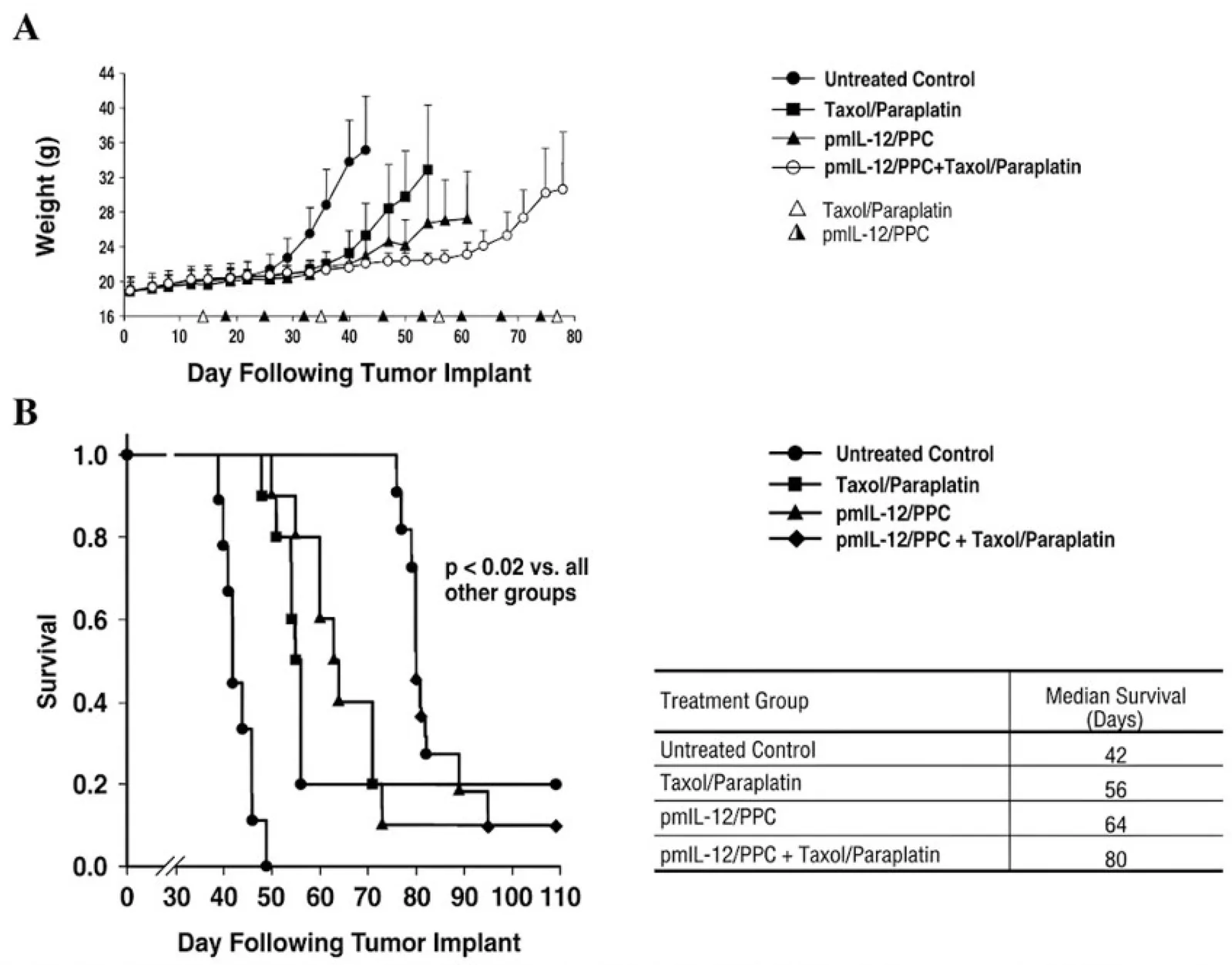
Antitumor efficacy of mIMNN-001 alone or in combination with paclitaxel/carboplatin in ID8 ovarian tumor-bearing mice. mIMNN-001 was administered once weekly for nine treatments, and paclitaxel/carboplatin was administered once every 3 weeks for four treatments. (**A**) Animal weight changes across treatment cohorts over time. (**B**) Survival across treatment cohorts. Significance between cohorts was determined by Gehan–Breslow nonparametric analysis of variance (*n* = 10 per cohort). Reproduced with permission from [58] John Wiley and Sons (2009); permission conveyed through Copyright Clearance Center, Inc.

**Table 1:** Long-term Stability of IMNN-001 at -20 °C Storage.

Test	Acceptance Criteria	0	3	6	9	12	24	36
Appearance—lyophilized	White cake	Conforms						
Appearance—post reconstitution	Clear, colorless to slightly milky white or slightly blue hued solution with no visible particulates	Conforms						
DNA Concentration (mg/mL)	0.5 mg/mL ± 0.15	0.49	0.50	0.50	0.50	0.49	0.53	0.5
Moisture (%)	≤ 2.5%	0.7	0.9	0.8	1.1	0.9	1.0	0.7
pH	3.0 ± 1.5	3.1	3.1	3.2	3.1	3.1	3.1	3.0
Osmolality (mOsm/kg)	300 mOsm/kg ± 50	310	301	306	303	300	299	306
Particle Size (nm)—Zavg	140 ± 30	138	138	137	138	139	137	137
Particle Size (nm)—D(10)	75 ± 25	72	74	71	76	74	73	73
Particle Size (nm)—D(50)	160 ± 30	162	159	161	157	161	156	158
Particle Size (nm)—D(90)	325 ± 75	355	343	368	336	354	360	349
Bioassay (%)	60%–140%	95	92	96	95	88	97	92
Reconstitution Time (sec)	< 120	33	32	41	39	37	32	40

**Table 2:** Summary of IL-12 delivery strategies designed to improve therapeutic safety.

Approach	Safety Rationale	Representative Examples	Key Limitations	IMNN-001 Differentiation
Recombinant IL-12 protein	Direct dosing can be stopped rapidly if toxicity occurs.	Systemic and historical intraperitoneal recombinant IL-12.	Short half-life, high systemic peaks, inflammatory toxicity, desensitization, and limited sustained tumor exposure.	Produces IL-12 locally for several days after regional plasmid administration.
Tumor-targeted immunocytokines or fusion proteins	Targeting and Fc engineering may enrich tumor exposure and reduce peak excursions.	NHS-IL12/M9241; engineered IL-12-Fc constructs.	Systemic administration, incomplete targeting, prolonged exposure, and residual peripheral activation.	Uses anatomic compartment targeting rather than antibody-mediated tumor targeting.
IL-12 mRNA delivery	Cytoplasmic translation enables finite expression without nuclear entry.	MEDI1191; self-replicating RNA-LNPs; organ-targeted and inhaled formulations.	RNA instability, innate sensing, off-target expression, carrier biodistribution, repeat-dose tolerability, and lesion access.	Can expose disseminated peritoneal disease without lesion-by-lesion injection.
Physical plasmid delivery	Local electroporation restricts plasmid uptake and IL-12 expression to injected lesions.	Tavokinogene telseplasmid with intratumoral electroporation.	Requires accessible lesions, specialized equipment, repeated procedures, and individual tumor injections.	Non-electroporation regional infusion provides compartment-wide exposure and repeat dosing.
Viral vector IL-12 gene delivery	Efficient local transduction and regulatable expression may reduce systemic cytokine exposure.	Replication-deficient adenoviral and regulatable IL-12 vectors.	Anti-vector immunity, inflammation, pre-existing immunity, shedding, manufacturing complexity, and re-dosing constraints.	Non-viral design avoids viral-vector immunity and supports repeated administration.
IL-12–armed oncolytic viruses	Tumor-selective replication couples oncolysis, antigen release, and local IL-12 production.	HSV-, adenovirus-, and vaccinia-based IL-12 oncolytic platforms.	Variable tumor permissiveness, antiviral immunity, inflammatory toxicity, payload stability, shedding, manufacture, and direct-injection requirements.	Does not depend on viral replication or tumor infectability and permits scheduled repeat dosing.
Extracellular-vesicle or exosome delivery	Biologically derived membranes may protect cargo and support route-specific tissue delivery.	Inhalable extracellular-vesicle IL-12 mRNA for lung delivery.	Early development; heterogeneous vesicles, loading and purification challenges, potency assays, storage, biodistribution, and batch consistency.	Uses a defined synthetic carrier with established cGMP manufacture, stability, catheter compatibility, and ovarian-cancer clinical experience.
Regional non-viral plasmid delivery	Transient, repeatable expression can localize cytokine activity while limiting systemic peaks.	IMNN-001 plasmid/PPC nanoparticles.	Requires intraperitoneal catheter access; local pain or inflammation; non-tumor-specific local transfection; nuclear entry required.	Compartment-wide ovarian-cancer delivery, repeatability, multi-day pharmacodynamics, and advanced clinical development.

## Data Availability

All data described in the review article may be obtained from the corresponding author upon reasonable request.
